# The Ethnopharmacology, Phytochemistry and Bioactivities of the *Corymbia* Genus (Myrtaceae)

**DOI:** 10.3390/plants12213686

**Published:** 2023-10-25

**Authors:** Matthew J. Perry, Phurpa Wangchuk

**Affiliations:** 1 College of Public Health, Medical and Veterinary Science, James Cook University, Cairns, QLD 4878, Australia; phurpa.wangchuk@jcu.edu.au; 2 Australian Institute of Tropical Health and Medicine, James Cook University, Cairns, QLD 4878, Australia

**Keywords:** biological activity, *Corymbia*, drug discovery, essential oils, ethnopharmacology, Myrtaceae, natural products, phytochemistry

## Abstract

Plants have been vital to human survival for aeons, especially for their unique medicinal properties. Trees of the *Eucalyptus* genus are well known for their medicinal properties; however, little is known of the ethnopharmacology and bioactivities of their close relatives in the *Corymbia* genus. Given the current lack of widespread knowledge of the *Corymbia* genus, this review aims to provide the first summary of the ethnopharmacology, phytochemistry and bioactivities of this genus. The Scopus, Web of Science, PubMed and Google Scholar databases were searched to identify research articles on the biological activities, phytochemistry and ethnomedical uses of *Corymbia* species. Of the 115 *Corymbia* species known, 14 species were found to have ethnomedical uses for the leaves, kino and/or bark. Analysis of the references obtained for these 14 *Corymbia* spp. revealed that the essential oils, crude extracts and compounds isolated from these species possess an array of biological activities including anti-bacterial, anti-fungal, anti-protozoal, anti-viral, larvicidal, insecticidal, acaricidal, anti-inflammatory, anti-oxidant, anti-cancer and anti-diabetic activities, highlighting the potential for this under-studied genus to provide lead compounds and treatments for a host of medical conditions.

## 1. Introduction

Over the past century, pharmaceutical interventions have become increasingly important in the treatment of ailments around the world, particularly in more developed nations. This is reflected in the ever-increasing investment of the pharmaceutical industry into drug research and development, which is reported to have increased from $2.3 billion USD in 1981 to $83 billion USD in 2019 [1]. This trajectory is unlikely to change anytime soon given the increasing prevalence of resistance to anti-microbials [2], and the need for more treatments to deal with the continual rise of conditions such as metabolic disorders and autoimmune diseases [3,4]. In less developed nations, however, access to pharmaceutical treatments is still limited, and as such, they continue to rely heavily upon medicinal plants for the treatment of many ailments [5].

For millennia, plants have been utilised by native cultures across the world for food, shelter, livelihood and medicine [6]. Even today, it is estimated that 65–80% of the world′s population continues to rely upon natural remedies due to a lack of access to modern medicine [7]. Ironically, it is to the native populations of the world who lack access to modern medicine that many researchers have been turning for inspiration and direction. Ethnopharmacology is becoming increasingly prevalent as a means of discovering new drug leads, as indigenous populations′ knowledge of plant medicinal properties can be utilised to direct the search for bioactive compounds [8]. The popularity of ethnopharmacological drug discovery is unsurprising given that approximately 40% of all small molecule therapeutics are natural products or derived from natural product pharmacophores [9] and that many of the over 50,000 medicinal plants known worldwide have not been screened for bioactive compounds to this day [7,10]. This is especially true of the many medicinal plants endemic to Australia.

Indigenous (aboriginal) Australians have lived from the land for thousands of years and have an intimate connection to and knowledge of endemic flora and their medicinal properties. Trees of the *Eucalyptus* genus (Myrtaceae) represent perhaps one of the most renowned Australian aboriginal bush medicines. These species are well-known for their volatile essential oils (EOs) which are extracted from the leaves and used to treat respiratory infections and inflammatory conditions around the world [11]. Further, *Eucalyptus* trees, while endemic to Australia, have been cultivated around the world and have become essential medicinal plants for other native populations around the world [12,13].

Despite the extensive knowledge and fame of the *Eucalyptus* genus for its medicinal properties, comparatively little is known about species of the *Corymbia* genus, which have similar phytochemical and medicinal properties [14]. The *Corymbia* genus comprises bloodwood, spotted and ghost gum trees, which were previously classified as subspecies of the *Eucalyptus* genus. In 1995, however, DNA and morphological research concluded that bloodwood, spotted and ghost gum trees were genetically distinct from other *Eucalyptus* species, and they were, therefore, reclassified as members of the *Corymbia* genus of the Myrtaceae family [15,16]. One key morphological characteristic of many *Corymbia* spp. is their production of kino, a resinous exudate which is used to treat many ailments by the aboriginal peoples of Australia [17]. Along with the known ethnomedical uses of various *Corymbia* species, a broad range of biological activities are observed in the EOs, crude extracts and compounds isolated from this genus [18,19,20,21], highlighting the potential of the *Corymbia* genus to provide new drug leads and treatments for many common diseases. To date, the ethnopharmacology, phytochemistry and biological activities of the *Corymbia* genus have not been reviewed, and it is therefore the aim of this study to provide the first ethnopharmacological summary for this genus and outline the biological activities of *Corymbia* spp. EOs, extracts and isolated compounds.

## 2. Literature Review Methods

Literature searches were initially performed using “*Corymbia*” as the keyword in the Scopus, PubMed and Web of Science databases. These references were filtered to include only journal articles, and the topics were limited to medicine, chemistry and pharmacology/pharmaceuticals in each database. Since *Corymbia* as a distinct genus classification has only existed since 1995 [15], all journal articles referring to these species were previously reported as *Eucalyptus* spp. Therefore, the names of the 115 known *Corymbia* species were identified using the World Flora Online database (https://www.worldfloraonline.org/, accessed on 4 May 2023) and each species was searched in Scopus as the equivalent “*Eucalyptus* spp.” to find journal articles related to these species prior to 1995. Combination of the references from each database search and removal of any duplicates provided 323 references. An additional four references related to the ethnomedical uses of *Corymbia* spp. were found through Google Scholar searches using the search terms “Australian aboriginal medicinal plants ethnopharmacology”. Of these 327 references, 105 references were determined to be relevant to the ethnopharmacology and biological testing of *Corymbia* spp.

## 3. Ethnomedical Uses of *Corymbia* Species

**Figure 1:**
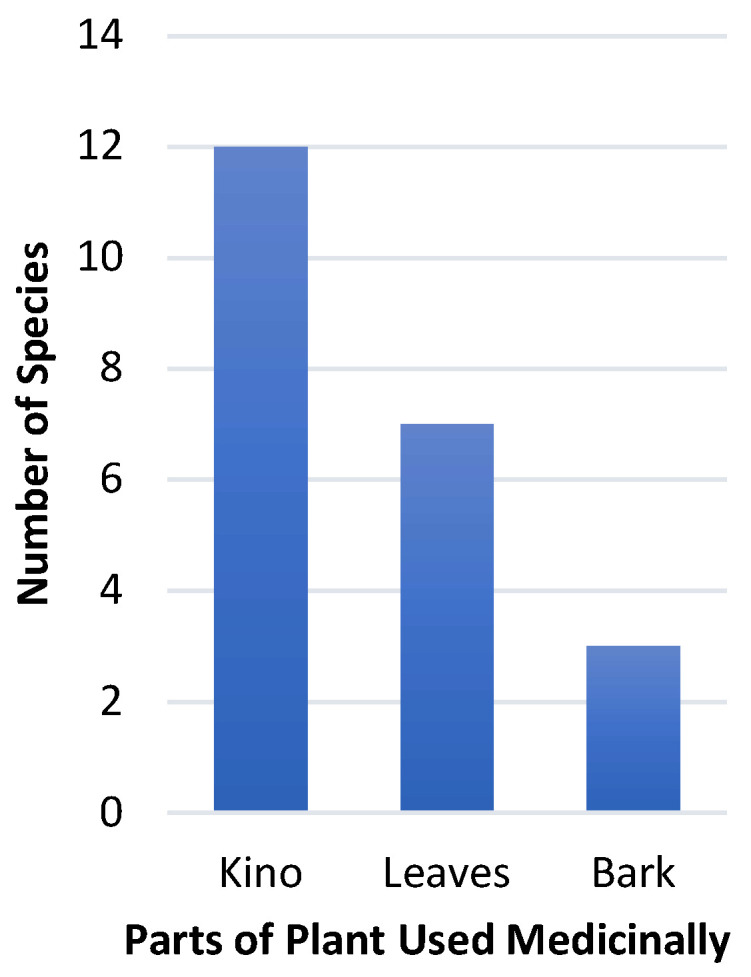
The use of plant parts in ethnomedically utilised *Corymbia* species (N = 14).

The ethnopharmacological data for the *Corymbia* genus presented in Table 1 show that of the 115 species known, ethnomedical uses have only been reported for 14 of these species. Of those 14 species, the kino was used medicinally in 12 spp., followed by the leaves (7 spp.) and bark (3 spp., Figure 1).

The kino of *Corymbia* genus plants is commonly applied directly to cuts (haemostatic), burns and wounds by aboriginal people and is added to water to make antiseptic washes [17,22,92,93,94]. Kino is applied locally to treat infections such as ringworm, venereal sores and other STIs [17,91] and is also ingested to treat pulmonary and heart complaints, gastrointestinal and bladder infections, diarrhoea and dysentery [17,40,41,89]. The kino of *C. terminalis* is also used as a tonic to treat blood conditions and to relieve headaches [22].

Hot water extracts of *Corymbia* spp. leaves are frequently used by aboriginal people as antiseptics for wounds and infections, analgesic baths for rheumatism and are ingested to treat respiratory and urinary tract infections and severe diarrhoea [28,29,30,31,32,33,34,35,90]. The leaves of *C. citriodora*, *C. maculata* and *C. torelliana* have also been adopted by the native populations of Cote d′Ivoire, Nigeria, India and Brazil for the treatment of toothaches, obesity and diabetes, respiratory and intestinal complaints, skin conditions, cancer, typhoid fever and malaria [28,30,34,35,36,37,38,111].

The less commonly used barks of the *Corymbia* spp. also possess medicinal properties. Gum derived from the bark of *C. maculata* is used in Australian bush medicine to treat bladder infections [30], while the bark of *C. terminalis* is used by aboriginal communities in Queensland to treat dysentery [91]. The bark of *C. citriodora* is also reported to be used in Nigeria as an antiseptic and expectorant and as a treatment for toothaches, diarrhoea and snake bites [29].

## 4. The Phytochemistry of *Corymbia* Species

### 4.1. Essential Oils

The EOs of *Corymbia* species have been of significant interest for many years, with their chemical composition typically quantified through metabolomic studies using GC–MS. Like *Eucalyptus* EOs, the EOs of *Corymbia* species are complex mixtures composed primarily of monoterpenoids and sesquiterpenoids, which exhibit a broad spectrum of biological activities. The exact chemical composition varies significantly between different species and within species according to the local climatic conditions, soil and the part of the plant [85,105,106,119].

The major constituents of various *Corymbia* spp. leaf EOs are summarised in Table 2. The leaf EO of *C. citriodora* is the most-studied within the genus [18,28,33,36,56,58,60,62,66,67,68,72,74,76,77,78,79,81,82,105,106,119,120,121,122,123,124,125,126,127,128,129,130,131,132,133,134,135,136,137,138,139,140,141,142,143,144,145,146,147,148,149,150,151], containing citronellal and citronellol as the major constituents, which have garnered significant attention for their various bioactivities (see Section 5) [152,153]. The bioactive monoterpenes α-pinene and β-pinene [154] have been observed to be key monoterpenoids in the leaf EOs of many *Corymbia* species, including *C. eximia*, *C. intermedia*, *C. maculata*, *C. polycarpa* and *C. torelliana*. Both *C. bleeseri* and *C. gummifera* produce bicyclogermacrene and β-caryophyllene as the key components of their leaf EOs. Distinct major constituents are also observed in the leaf EOs of *C. calophylla* (γ-terpinene and (*E*,*E*)-farnesol) and *C. tessellaris* (aromadendrene and globulol), highlighting the extreme variability observed in leaf EO compositions.

In addition to leaf EOs, the EOs of flowers, twigs and fruits have also previously been studied in *C. citriodora* and *C. torelliana* (Table 3). The major constituents of *C. citriodora* twig EO have been observed to be 1,8-cineole and *p*-cymene, while the fruit EO has been reported to mainly comprise α-pinene and γ-terpinene. The flower EO of *C. citriodora* has also been observed to contain α-pinene as the major terpenoid (54.1%), along with citronellol as a minor component (9.9%). In *C. torelliana*, α-pinene is observed to be the major constituent in both leaf and flower EOs, though differences are observed in the minor components of these EOs (β-caryophyllene and β-pinene, respectively). This variability in EO composition observed both between species and in different parts of the plant studied warrants further investigation and review as this presents an excellent opportunity for commercialisation as alternatives to traditional *Eucalyptus* oils, as these mixtures could be marketed according to the key bioactivities observed in the major components of these EOs.

### 4.2. Isolated Natural Products

Analysis of the compounds reported in Table 1 revealed that 147 distinct molecules have previously been isolated from *Corymbia* species, with the major classes of phytochemicals summarised in Table 4 below. Species of the *Corymbia* genus are rich in polyphenols, particularly flavonoids, which account for the potent anti-inflammatory properties observed in crude extracts (see Section 5.3). Terpenoids are also key metabolites isolated from *Corymbia* species, in particular, triterpenoids, in addition to the hundreds of monoterpenoids and sesquiterpenoids detected in the EOs of these species. Twenty polyketides have also been previously isolated, including 16 β-triketones from *C. intermedia* and *C. torelliana* which showed potent anti-protozoal activity (see Section 5.1.3). The chemical structures of key bioactive compounds previously isolated are presented throughout Section 5.

## 5. Overview of Biological Testing in *Corymbia* Species

**Figure 2:**
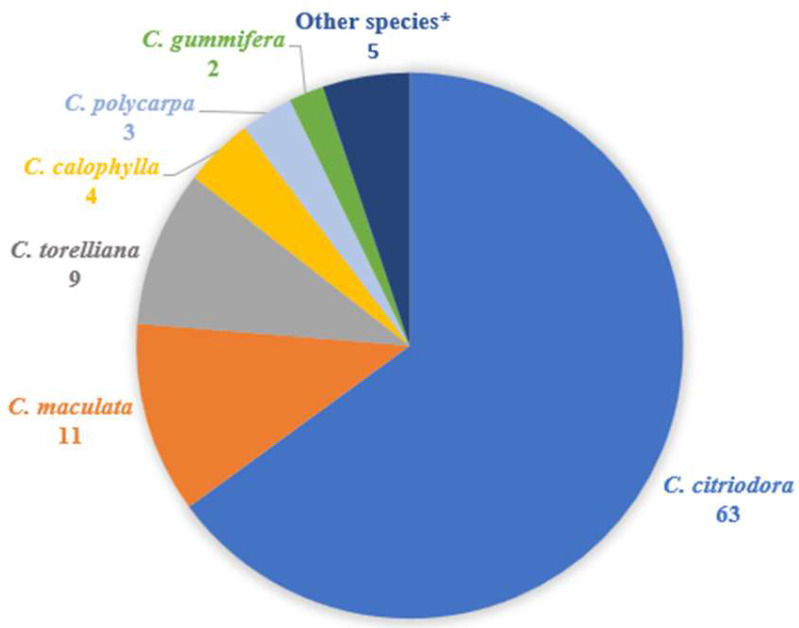
A comparison of the number of research articles reported on *Corymbia* species. Other species* refers to *C. blesseri*, *C. eximia*, *C. intermedia*, *C. papuana*, *C. terminalis* and *C. tessellaris* which had one reference each. No publications were reported for *C. dichromophloia* and *C. opaca* (excluding ethnomedical uses).

**Figure 3:**
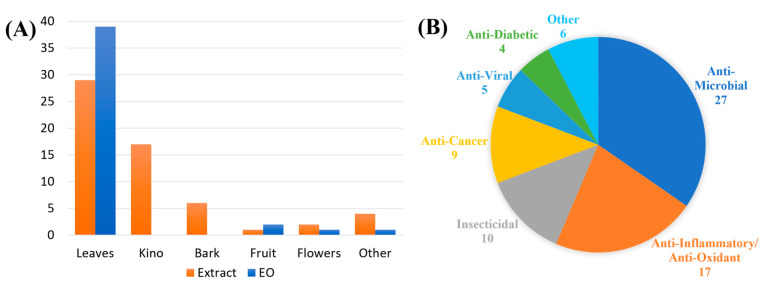
The number of references reported for ethnomedically significant *Corymbia* species, analysed according to (**A**) plant part studied and (**B**) biological activities investigated.

Analysis of the 105 publications reported for *Corymbia* spp. (excluding purely ethnopharmacological studies) in Table 1 revealed that *C. citriodora* was the most-studied species (N = 63), followed by *C. maculata* (N = 11), *C. torelliana* (N = 9) and *C. calophylla* (N = 4), highlighting the dearth of study into most species of this genus (Figure 2). Further analysis of references reporting biological activities revealed that the leaf Eos and leaf extracts of *Corymbia* species were the most commonly studied (N = 68), followed by kino (N = 17) and bark (N = 6) extracts (Figure 3A). When analysed according to biological activity, anti-microbial and anti-viral activities were found to account for almost half of the references (N = 32), followed by anti-inflammatory/anti-oxidant (N = 17), anti-cancer (N = 8) and insecticidal (N = 7) activities (Figure 3B). These biological activities are discussed in the subsections below.

### 5.1. Anti-Microbial and Anti-Viral Activity

#### 5.1.1. Anti-Fungal Activity

The leaf EO of *C. citriodora* has been established to have excellent anti-fungal activity and has been observed to have low microgram to high nanogram/mL MIC and MFC concentrations in a wide variety of species including *C*. *albicans*, *C*. *krusei*, *C*. *tropicalis*, *A. alternata*, *C. lunata*, *B. specifera*, *M. canis*, *M. gypseum*, *T. mentagrophytes, T. rubrum*, *A. flavus*, *A. ochraceus*, *A. niger*, *F. oxysporum*, *P. funiculosum*, *P. ochrochloron* and *R. solani* [33,36,55,56,57]. The major constituent of *C. citriodora* EO, citronellal [33,56], has previously been shown to have potent anti-fungal activity against *R. solani* and *H. oryzae* [156]. The fruit EO of *C. citriodora* and two key components of this mixture (τ-cadinol and α-cadinol) can also inhibit the growth of *A. clavatus*, *A. niger*, *Cl. Cladosporioides*, *Ch. Globosum*, *M. verrucaria*, *P. citrinum* and *T. viride* [60]. Recently, *C. citriodora* leaf EO and citronellol acetate (a minor constituent of the EO) were shown to effectively treat and enhance the rate of healing of rats with *C. albicans*-infected wounds [44]. The enhanced wound healing observed in rats treated with the EO was suggested to be the result of the presence of α-pinene, which promotes collagen formation, deposition and maturation [44,157].

**Figure 4:**
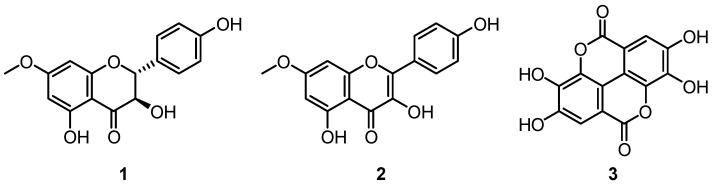
Three flavonoids isolated from the kino of *C. citriodora* which exhibited anti-fungal activity [54].

Extracts of *C. citriodora* have been studied to a lesser degree than leaf EOs, but they have been shown to possess anti-fungal activity. The petroleum ether leaf extract of *C. citriodora* has been shown to possess moderate inhibitory and fungicidal activity against *A. flavus* and *A. parasiticus* [58]. Compounds, including 7-*O*-methylaromadendrin **1**, 7-*O*-methylkaempferol **2** and ellagic acid **3,** isolated from the kino of *C. citriodora* (Figure 4) have also been shown to have varying anti-fungal activities against *P. notatum*, *A. niger* and *F. oxysporium* [54].

#### 5.1.2. Anti-Bacterial Activity

Given the anti-fungal activity observed in the EO of *C. citriodora*, it is unsurprising that it also exhibits broad spectrum anti-bacterial activity. Inhibitory and bactericidal activity have been reported against *A. tumefaciens*, *B. cereus*, *D. solani*, *E. coli*, *P. atrosepticum*, *P. carotovorum* and *S. aureus* [33,36]. Thirty-two components of the *C. citriodora* leaf EO have been identified as being able to inhibit airborne *M. tuberculosis* [62], while anti-bacterial and possible anti-biofilm activity have been observed against various strains of *S. sanguinis* and *S. salivarius* [18]. Twig and fruit EOs from *C. citriodora* have also been shown to inhibit *B. cereus*, *S. aureus*, *E. aerogenes*, *K. pneumoniae*, *P. aeruginosa*, *V. parahaemolyticus*, *S. epidermidis* and *E. coli* bacteria with MIC values ranging from 125 to 1000 µg/mL [60]. The leaf EO of *C. citriodora* has also been shown to inhibit the growth of *V. campbelli* and was successfully used to treat gnotobiotic brine shrimp (*Artemia franciscana*) infected with *V. campbellii* and enable their survival [63,64].

Petroleum ether, MeOH and EtOH leaf extracts of *C. citriodora* have also demonstrated moderate anti-bacterial activity against a host of gram-positive and gram-negative bacteria, including *E. faecalis*, *S. aureus*, *A. baumannii*, *C. freundii*, *E. aerogenes*, *E. coli*, *K. pneumoniae*, *P. mirabilis*, *P. aeruginosa*, *B. subtilis*, *P. fluorescens* and *V. parahaemolyticus* [37,59,61]. Anti-bacterial activity has also been reported in various kino extract fractions against *M. aureus* and *My. pheli* [54].

**Figure 5:**
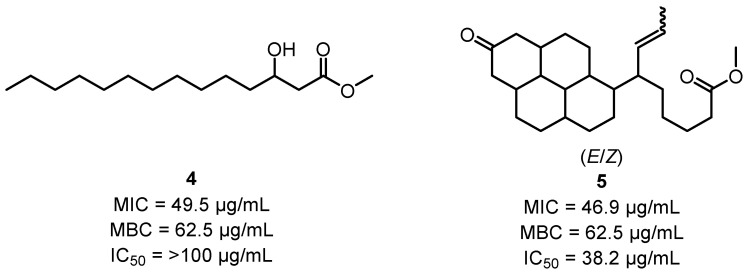
Anti-tuberculosis activity of hydroxymyristic acid methyl ester **4** and methyl (*E*)- and (*Z*)-6-(8-oxooctadecahydrochrysen-1-yl)non-7-enoate **5** against *M. tuberculosis* H37RvATCC 27294 [115].

Research over recent years has also shown the anti-bacterial potential of *C. torelliana*. Moderate anti-bacterial activities were observed for the leaf and fruit EOs of *C. torelliana* against *B. cereus*, *S. aureus*, *E. coli*, *P. aeruginosa*, *C. albicans* and *A. niger* [111]. Leaf extracts of *C. torelliana* have been shown to have potent inhibitory activity against several strains of *H. pylori* [110], while crude propolis derived from the kino of *C. torelliana* and *C*-methyl flavones isolated therefrom exhibited bactericidal activity against *S. aureus* [116]. Hydroxymyristic acid methyl ester **4** and methyl (*E*)- and (*Z*)-6-(8-oxooctadecahydrochrysen-1-yl)non-7-enoate **5** isolated from the leaves of *C. torelliana* (Figure 5) also exhibited bactericidal activity against *M. tuberculosis* [115].

#### 5.1.3. Anti-Protozoal Activity

The leaf EO of *C. citriodora* has been shown to have anti-trypanosomal activity against *T. brucei*, *T. evansi* and *T. cruzi* [67,68], while crude EtOH leaf extracts have shown potent inhibitory activity against *T. brucei* and *P*. *falciparum* 3D7 and INDO strains [69,70].

**Figure 6:**
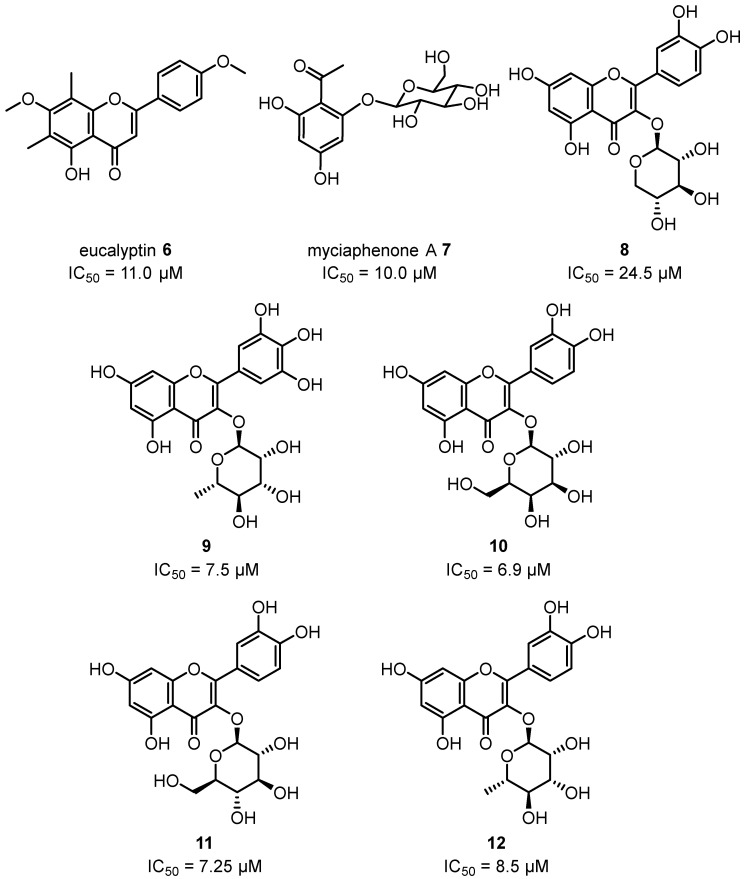
Flavonoids and flavonoid glycosides isolated from the leaves of *C. maculata* and their anti-leishmanial activity against *L. donovani* [96].

Crude extracts and one isolated compound from the leaves of *C. maculata* showed inhibitory activity against *T. brucei* [101]. Eucalyptin **6**, myciaphenone A **7** and flavonoid glycosides **8**–**12** (Figure 6) isolated from the leaves of *C. torelliana* have also shown potent anti-leishmanial activity against *L. donovani* [96]. A comprehensive study of the biological activities of over 100 flavonoids and polyphenols against *Leishmania donovani*, *Trypanosoma brucei rhodesiense* and *Trypanosoma cruzi* has been previously reported; however, despite these efforts, clear quantitative structure–activity relationships (SARs) could not be established [158]. More recent work has shown that various flavonoid glycosides, including quercetin-3-*O*-β-D-galactoside **10**, inhibit *Leishmania amazonensis* arginase [159], which may represent an important mode of action for the anti-protozoal activity observed in flavonoid glycosides and could be a target for further drug lead development and SAR optimisation.

**Figure 7:**
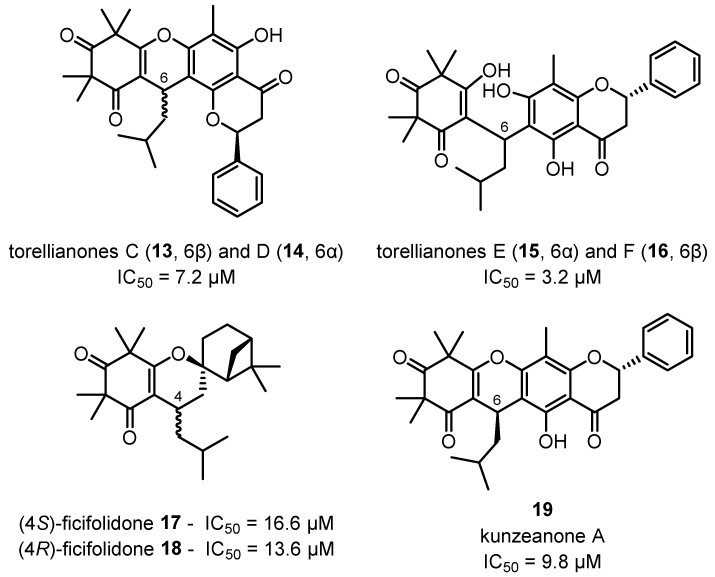
Torellianones C-F **13**–**16**, ficifolidones **17**–**18** and kunzeanone A **19** isolated from the flowers of *C. torelliana* and their anti-plasmodial activities against *P. falciparum* [112].

**Figure 8:**
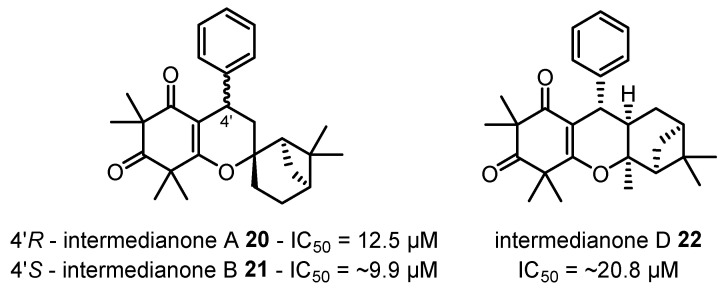
Intermedianones A, B and D **20**–**22** isolated from the flowers of *C. intermedia* and their anti-plasmodial activities against *P. falciparum* 3D7 [19].

A new class of anti-plasmodial β-triketones has also been discovered in recent years, with micromolar inhibition of *P. falciparum* observed in torellianones C–F **13**–**16**, ficifolidones **17**–**18**, kunzeanone A **19** (Figure 7) and intermedianones A, B and F **20**–**22** (Figure 8) isolated from the flowers of *C. torelliana* and *C. intermedia*, respectively [19,112]. Three additional anti-plasmodial β-triketones, watsonianones A–C, have also been previously isolated from the flowers of *C. watsoniana* (F. Muell.) [160]. Although no molecular targets have been identified for these β-triketones at this stage, these results emphasise the pharmacological potential for *Corymbia* species to provide new lead compounds for the treatment of significant diseases.

#### 5.1.4. Anti-Viral Activity

Very few studies into the anti-viral activities of *Corymbia* species have been published. A molecular docking study suggested that several components of the EO of *C. citriodora*, particularly 1,8-cineol and α-pinene, could potentially inhibit the M^Pro^ protein of SARS-CoV-2 [139]. Further research revealed that citronellal (the major component of *C. citriodora* EO) and 1,8-cineol are inhibitors of ACE2 and LOX, suggesting the EO may have potential for use as an anti-viral and anti-inflammatory agent [81].

**Figure 9:**
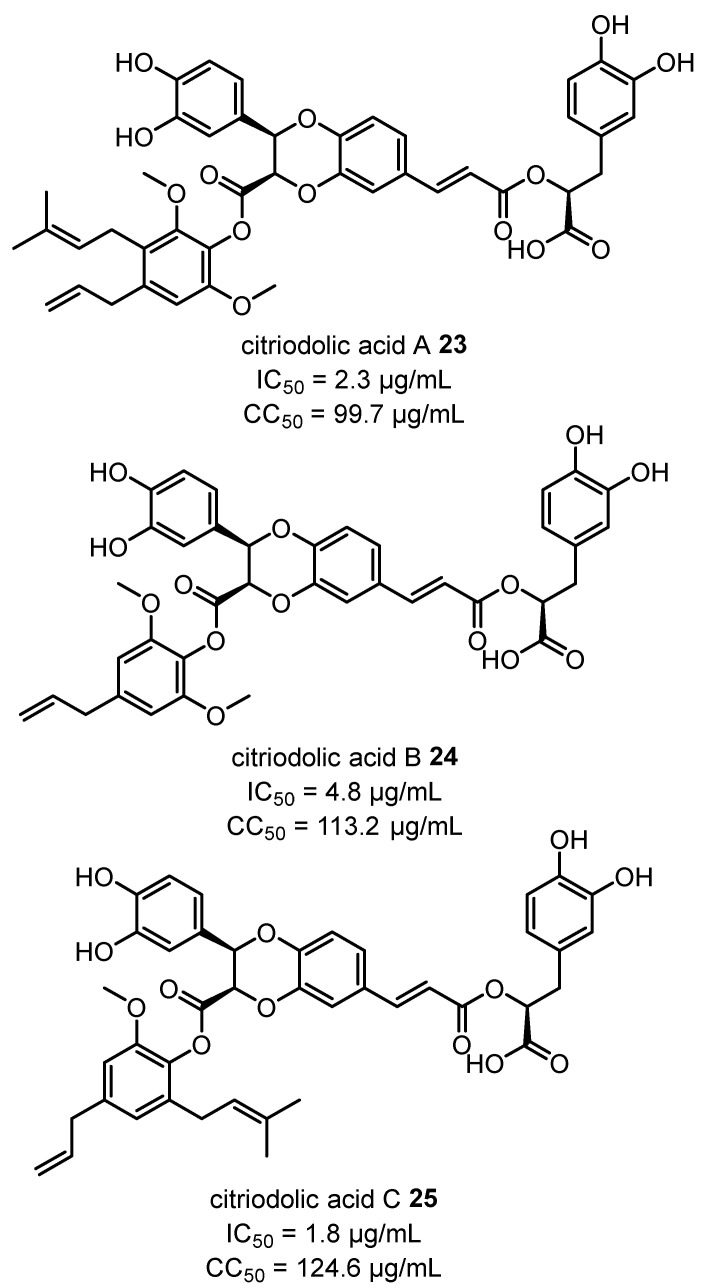
Citriodolic acids A–C **23**–**25** isolated from the leaves of *C. citriodora* and their anti-viral activities against RSV [50].

**Figure 10:**
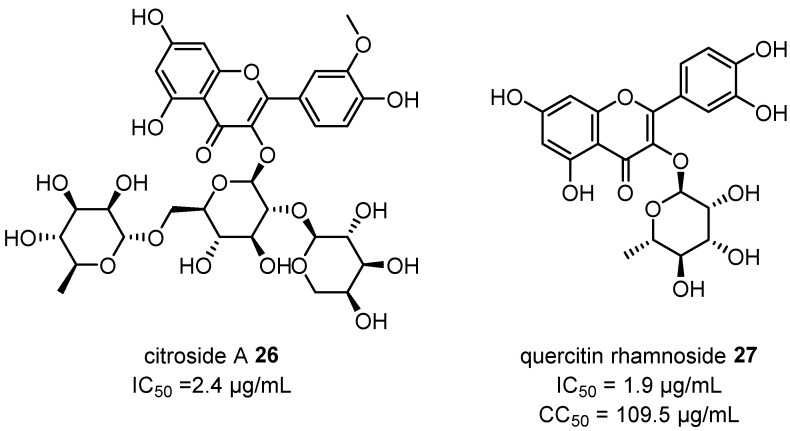
Citrioside A **26** and quercetin-3-*O*-α-L-rhamnoside **27** isolated from the leaves of *C. citriodora* and their anti-viral activities against RSV [52,53].

Significantly more work has been performed with an eye toward isolating anti-viral compounds against respiratory syncytial virus (RSV) in the laboratory of Zhong-liu Zhou. Citriodolic acids A–C **23**–**25** (Figure 9) were isolated from the EtOH extract of *C. citriodora* leaves and showed potent activity against RSV (IC_50_ = 1.8–4.8 µg/mL) comparable to that reported for ribacirivin, a drug already approved for the treatment of RSV infections [50]. Further extractions and isolations of *C. citriodora* leaves also yielded citrioside A **26** and quercetin-3-*O*-α-L-rhamnoside **27**, which also showed potent activity against RSV (Figure 10). Although further research is required to elucidate the modes of action for the anti-viral activity of these compounds, these results highlight the potential of *Corymbia* gum trees to provide new anti-viral lead compounds [52,53].

### 5.2. Insecticidal Activity

A variety of studies have been reported on the insecticidal activities of *Corymbia* species, mainly focussing upon the application of crude extracts and crude EOs. Larvicidal and acaricidal activity of the EO of *C. citriodora* has been reported in a variety of species, including *A. aegypti* mosquitoes [72], the brine shrimp *Artemia salina* [59], the fall armyworm *Spodoptera frugiperda* [74], the Asian blue tick *Rhipicephalus microplus* [66] and the tropical horse tick *Anocentor nitens* [65]. Insecticidal and fumigant activities have also been reported for the leaf EO of *C. citriodora* against the cabbage moth *Plutella xylostella* [150] and Japanese termite *Reticulitermes speratu* [71], respectively. The leaf EOs of *C. citriodora*, *C. maculata* and *C. torelliana* also exhibit moderate fumigant and repellent activity against coffee berry borer *Hypothenemus hampei* females [151]. Larvicidal activity has been reported for the hexane extract of *C. citriodora* leaves against *Anopheles stephensi*, *Culex quinquefasciatus* and *Aedes aegypti* mosquitos [73], while the MeOH extract is lethal to the red flower beetle *Tribolium castaneum* [75].

### 5.3. Anti-Inflammatory and Anti-Oxidant Activity

The anti-inflammatory and anti-oxidant activities of the *Corymbia* species are well established in the literature. Many publications on the EOs of *C. citriodora* report very low IC_50_ and high percent inhibition values in DPPH assays [31,33,76,77,78,79]. The floral EO of *C. citriodora* is reported to have more moderate anti-oxidant activities [31], while the aqueous extract of *C. citriodora* leaves and MeOH extract of *C. maculata* resin exhibited potent activities in DPPH assays [80,102].

The potent anti-inflammatory properties of the EO of *C. citriodora* have also been demonstrated further in vitro through the low µg/mL IC_50_ values obtained in β-Carotene-linoleic acid and protease inhibitory assays [31,33]. One study also found that the floral EO of *C. citriodora* exhibited potent protease inhibition with an IC_50_ = 2.59 µg/mL [31].

**Figure 11:**
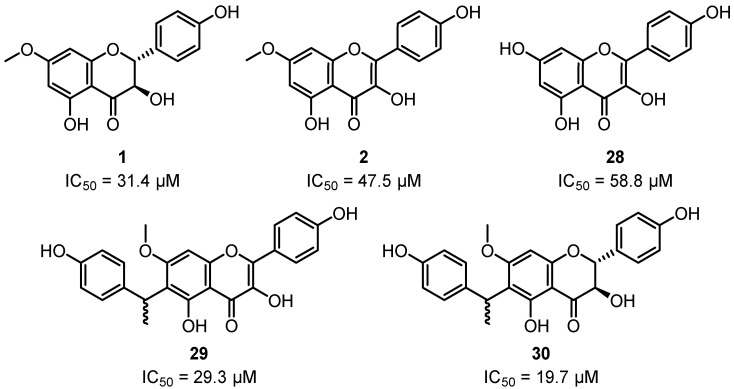
Flavanols isolated from the kino of *C. citriodora* and their in vitro inhibitory activities against 15-LOX [49].

The leaf EO of *C. citriodora* and its constituents citronellal and 1,8-cineol have been observed to inhibit ACE2 and LOX enzymes in vitro, suggesting there are multiple anti-inflammatory modes of action [81]. Additional studies have also shown that the leaf EO and resin extracts of *C. citriodora* inhibit LOX-1 and 15-LOX, respectively [28,32]. Extraction and purification of the EtOAc fraction of the EtOH extract of *C. citriodora* kino led to the isolation of 7-*O*-methylaromadendrin **1**, 7-*O*-methylkaempferol **2** and flavonoids **28**–**30** (Figure 11), which were also shown to inhibit 15-LOX [49].

In a cell-based in vitro assay of LPS-induced RAW264.7 macrophages, the leaf EO of *C. citriodora* showed anti-inflammatory effects (reduced levels of NO, IL-6, TNF-α, COX-2 and iNOS expression) through the inhibition of MAPK and NF-κB pathways [82]. A similar study of LPS-induced RAW264.7 macrophages was performed on crude extracts of *C. gummifera*, *C. maculata* and *C. eximia*, which were shown to exert anti-inflammatory properties through the inhibition of NO and TNF-α production [90].

**Figure 12:**
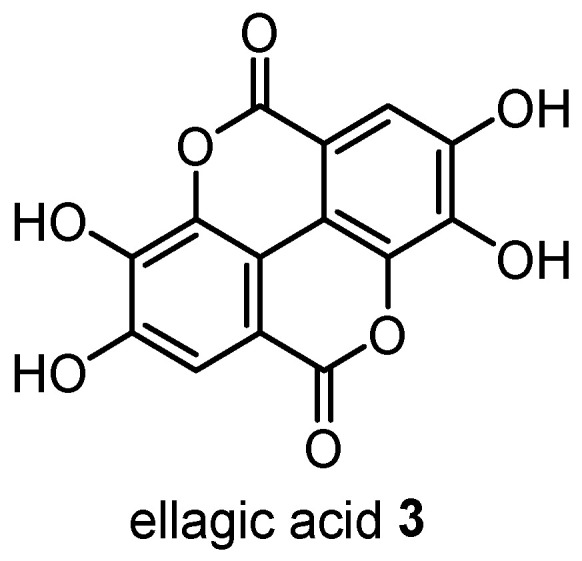
Ellagic acid **3**, isolated from the leaves of *C. citriodora*, exhibited potent anti-inflammatory and gastroprotective properties in an EtOH-induced gastric ulcer mouse model [39].

An in vivo investigation of the anti-inflammatory and analgesic activities of the leaf EO of *C. citriodora* was performed in rats and showed that the EO inhibits central and peripheral nociception, as well as neutrophil-independent and neutrophil-dependent inflammation [35]. Another in vivo investigation showed the ellagitannin-rich fraction extracted from the leaves of *C. citriodora* had anti-inflammatory and gastroprotective effects in EtOH-induced rats [20]. This research was expanded upon, wherein the ellagitannin ellagic acid **3** (Figure 12) was isolated from the leaves of *C. citriodora* and shown to have significant anti-inflammatory (increased IL-10 and PGE2 levels and decreased IL-6, TNF-α and COX-2 levels) and anti-gastric ulcer effects in EtOH-induced mice [39]. Analysis of the aspartate aminotransferase and creatine levels also showed little change between the control and ellagic acid **3** treatment groups, suggesting that ellagic acid has no adverse effect on liver and kidney function, highlighting the potential therapeutic value of this natural product [39].

**Figure 13:**
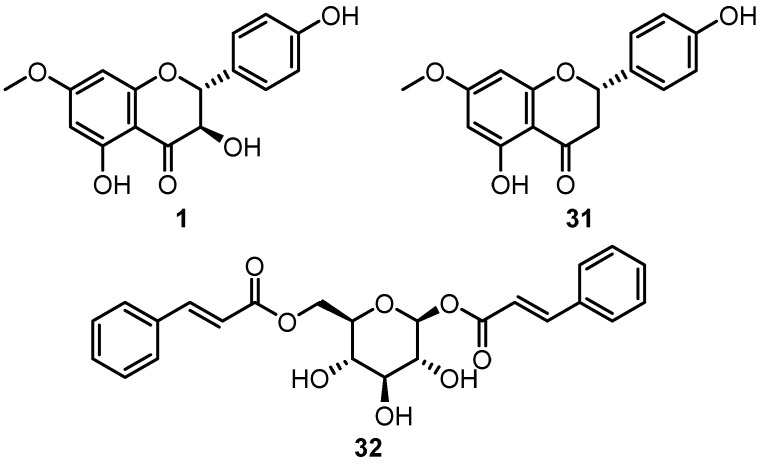
Flavonoids **1**, **31** and glucoside **32** isolated from the kino of *C. maculata* showed anti-oxidant and hepatoprotective properties in acetaminophen-induced mice [99,103].

The kino of *C. maculata* has also garnered some attention for its anti-inflammatory and anti-oxidant activities. The chloroform extract of the kino of *C. maculata* and isolated compounds 7-*O*-methylaromadendrin **1**, sakuranetin **31** and 1,6-dicinnamoyl-*O*-α-D-glucoside **32** (Figure 13) were shown to protect against acetaminophen lethality in rats and significantly reduced the rise in aspartate aminotransferase, alkaline phosphatase and alanine aminotransferase levels induced by acetaminophen [99,103]. More recently, the MeOH extract of *C. maculata* kino was shown to reduce levels of NF-κB, TNF-α, COX-2 and NO inflammatory biomarkers and significantly reduced paw thickness in carrageenan-induced paw oedema in rats [102].

### 5.4. Anti-Cancer Activity

The EOs and extracts of several *Corymbia* species have been shown to have cytotoxic and anti-proliferative effects in a range of different cancers. The EO of *C. citriodora* has been shown to exhibit anti-proliferative effects against leukaemia THP-1 cells [84] and cytotoxicity against lung cancer A-549, prostate cancer PC-3, glioblastoma T98G, breast cancers T47D and MCF-7, colon cancer HCT-116 and liver cancer Hep G-2 cells [57,76]. The fruit EO of *C. citriodora* also showed cytotoxic activity in A549, HeLa and CHOK1 cells [85]. In a like manner, the leaf and fruit EOs of *C. torelliana* were also observed to have cytotoxicity toward PC-3, Hep G2, Hs 578T and MDA-MB-231 cell lines [111].

**Figure 14:**
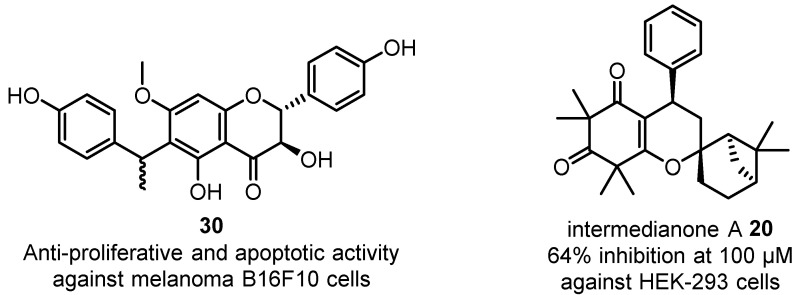
Anti-proliferative natural products flavonoid **30** isolated from *C. citriodora* kino [40] and intermedianone A **20** isolated from the flowers of *C. intermedia* [19].

Anti-proliferative effects have been observed in the aqueous extracts of *C. citriodora* and *C. maculata* leaves against PaCa-2 cells [30]. The aqueous fraction obtained from the EtOH extract of *C. citriodora* kino was observed to inhibit cell growth and induce apoptosis in Hep G2 cells [41]. The EtOAc kino extract of *C. citriodora* and its major constituent, flavonoid **30** (Figure 14), have shown anti-proliferative activity and cytotoxicity through apoptosis induction in B16F10 melanoma cells [19,40]. Novel β-triketone-monoterpene intermedianone A **20** isolated from the flowers of *C. intermedia* also exhibited anti-proliferative activity against HEK-293 cells [19]. Further investigations are required to elucidate the modes of action and molecular targets of these novel anti-cancer agents.

### 5.5. Anti-Diabetic Activity

**Figure 15:**
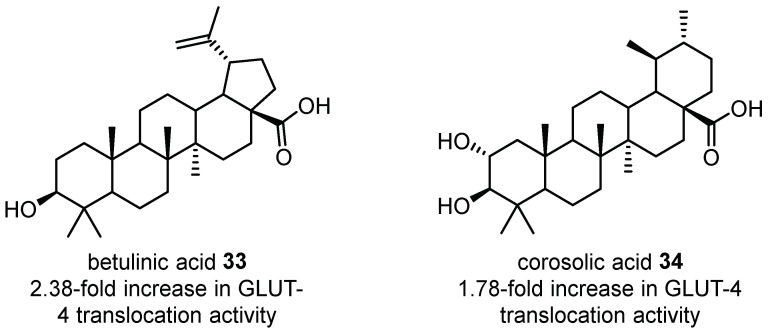
Betulinic and corosolic acids **33**–**34** isolated from the leaves of *C. citriodora* and their in vitro GLUT-4 translocation activities [51].

Very few studies have investigated the anti-diabetic properties of *Corymbia* species; however, recent studies have shown the leaves of *C. citriodora* may have potential for utility in this area. Betulinic acid **33** and corosolic acid **34** (Figure 15) isolated from the aqueous extract of *C. citriodora* were shown to enhance GLUT-4 translocation activity by 2.38- and 1.78-fold, respectively, in vitro [51]. Further research on the aqueous extracts of *C. citriodora* leaves has shown their ability to stimulate insulin production and glucose uptake in BRIN-BD11 clonal pancreatic β-cell cells, islets of Langerhans and in high-fat-fed rats [83]. Another study performed in alloxan-induced diabetic mice revealed that treatment with *C. citriodora* aqueous extracts significantly lowered blood glucose levels, comparable to the levels observed in the glibenclamide control [80]. Most recently, the EtOH extract of *C. citriodora* leaves was shown to have significant anti-diabetic and insulinotropic activity in BRIN-BD11 cells, islets of Langerhans and in a high-fat-fed rat model [21], underscoring the need for further research into the anti-diabetic potential of other *Corymbia* spp. and the molecular targets through which the *C. citriodora* aqueous extracts exert anti-diabetic activity.

### 5.6. Other Biological Activity

The extracts of *Corymbia* species have been observed to have a broad spectrum of bioactivities and effects. The EO of *C. citriodora* leaves is well known for its mosquito repellence activity, particularly through its major constituent, citronellal [87,161,162]. More recently, isomers of *p*-menthane-3,8-diol isolated from the leaves of *C. citriodora* have also been shown to have repellent activity against *A*. *gambiae* mosquitoes [43]. Various field trials investigating *C. citriodora* mosquito repellence have been performed using live plants [163], burning leaves [164,165], leaf EOs [166] and a mosquito repellent product (Quwenling) [167]; however, only moderate effectiveness has been observed. This lack of effectiveness is due to the volatility of citronellal and other mosquito-repellent components of the leaf EOs, which only protect individuals for the first hour of use [168]. Additionally, one field trial indicated that biting midges (*Culicoides imicola*) were attracted to test sites using a mosquito repellent based on *C. citriodora* [166].

A recent study into the aqueous extracts of *C. citriodora* leaves and branches showed they had the capacity to detoxify mycotoxins aflatoxins B_1_ and B_2_, both in vitro and in vivo in brine shrimp (*Artemia salina*) larvae [86]. The acetone leaf extract of *C. citriodora* has also been observed to significantly delay the loss of climbing ability and reduce oxidative stress in transgenic *Drosophila* expressing h-αS in the neurons, suggesting that *Corymbia* spp. could have potential applications in the treatment of neurological diseases, such as Parkinson′s disease [88].

Given the ethnomedical use of *C. citriodora* leaves as a remedy for diarrhoea, the leaf EO of *C. citriodora* was tested and found to have significant anti-spasmodic effects in rats experiencing acetylcholine-induced contraction of the ileum [29]. Leaf and stem bark extracts of *C. torelliana* have also been shown to have gastroprotective and anti-secretory activities in rats induced with EtOH/HCl, illustrating the diversity of biological activities observed in *Corymbia* species and their potential for the discovery of new treatments for common ailments [118].

## 6. Conclusions and Future Directions

This review has provided the first summary of the ethnopharmacology, phytochemistry and biological activities of the *Corymbia* genus. Of the 115 species of the *Corymbia* genus, 14 species were found to have ethnomedical uses for the leaves, kino and bark. *Corymbia citriodora* was the most studied species, followed by *C. maculata* and *C. torelliana*. Outside of these three species, no more than four references on bioactivities were found for any of the other 112 species of the *Corymbia* genus, revealing the current dearth of study into these species and their potential medicinal uses.

The leaf EOs of *Corymbia* spp. were found to differ greatly in chemical compositions and exerted a broad spectrum of biological activities. The leaf EO of *C. citriodora* in particular was shown to have excellent antiseptic, anti-microbial, insecticidal, anti-inflammatory, anti-oxidant and anti-cancer properties, making this an ideal product for further development and commercialisation as a lemon-scented alternative to *Eucalyptus* oil. Due to the variability observed in leaf EO composition, further studies would be required to standardise these mixtures to ensure consistent chemical compositions are obtained. Additional research analysing the EOs from other unstudied *Corymbia* spp. and EOs from other parts of the plant could also be fruitful in the development of commercial products with more specific uses (e.g., insecticidal EOs, anti-septic EOs or anti-inflammatory EOs) based upon the major constituents of the EOs and their key bioactivities.

The crude extracts of *Corymbia* spp. were also reported to exhibit even more broad bioactivities than the EOs, providing another area for further investigation. Of particular note are the potent anti-diabetic effects observed in the aqueous extracts of *C. citriodora*, supporting the ethnomedicinal use of these extracts in treating diabetes. These promising preliminary results observed in multiple in vivo models warrant investigations in phase I clinical trials as a crude mixture and further isolation to identify additional anti-diabetic lead compounds.

The low micromolar anti-plasmodial activities observed for eucalyptin **6**, myciaphenone A **7** and flavonoid glycosides **8**–**12** against *L. donovani* and for torellianones C–F **13**–**16**, ficifolidones **17**–**18**, kunzeanone A **19** and intermedianones A, B and F **20**–**22** against *P. falciparum* emphasise the potential of *Corymbia* spp. as a source of anti-plasmodial lead compounds. Additional investigations are required to better define molecular targets and produce SARs for these anti-plasmodial lead compounds prior to preclinical trials.

The potent anti-viral activity of the novel compounds citriodolic acids A–C **23**–**25**, citrioside A **26** and quercetin-3-*O*-α-L-rhamnoside **27** (isolated from the leaves of *C. citriodora*) against RSV merit additional investigations to elucidate modes of action and determine treatment efficacy in vivo. Additional research for further lead compound discovery and screening for anti-viral activity in other common viral pathogens could also be invaluable avenues for further exploration.

Overall, this review has provided a preliminary summary of the ethnopharmacology, phytochemistry and bioactivities of the *Corymbia* genus, highlighting the potential for these species to provide lead compounds to treat a host of common medical conditions. Since the extraordinary bioactivities of less than one fifth of the 115 *Corymbia* spp. have been studied previously, the question is this: what else is waiting to be discovered?

## Figures and Tables

**Table 1:** Ethnopharmacological data for *Corymbia* spp. plants and biological testing performed.

Species	Origin of Plant Studied	Part(s) of Plant Studied ^§^	Ethnomedical Uses	Compounds Isolated	Biological Testing Performed
*C. bleeseri* (Blakely)	Australia	K	Kino is applied to cuts and wounds to promote healing [22] and is used to treat skin lesions, scabies, cramps, sore throats and coughs [17].	– † [23]	–
*C. calophylla* (Lindl.)	Australia	K, B	Kino is used to treat chronic bowel conditions and dysentery [17].	Aromadendrin; kaempferol; ellagic acid [24]; oleanolic acid acetate [25]; (+)-afzelechin *; pyrogallol; (+)-catechin [26]; leucopelargonidin *; aromadendrin; sakuranetin [27].	–
*C. citriodora* (Hook.)	Algeria; Australia; Bangladesh; Benin; Brazil; China; Colombia; Cote d′Ivoire; Democratic Republic of the Congo; Egypt; India; Türkiye; Kenya; Madagascar; Morocco; Nigeria; Pakistan; Portugal; Taiwan; Thailand; USA	L, K, T, Fr, H	Leaves and bark are used as antiseptics, expectorants, and treatments for influenza and colds, toothaches [28,29] and diarrhoea [29]; hot water extracts of the dried leaves are used to treat colds, influenzas, respiratory infections and sinus congestion [30,31,32,33,34,35]; water extracts are also used to treat, vomiting, nausea, indigestion, bloating, irritable bowel and abdominal pain [30]; leaves are used in India and Africa to treat obesity, ageing, cardiovascular illnesses, diabetes and respiratory problems [36,37]; in Nigeria, leaves are boiled and consumed for the treatment of typhoid fever, stomach aches and malaria [38]; Dharawal people use leaves to treat inflammation, wounds and fungal infections [39]. Kino is traditionally used to treat diarrhoea and bladder inflammation and is applied to cuts and abrasions [40,41].	Shikimic acid; quinic acid; glutaric acid; succinic acid; malic acid; citric acid [42]; (±)-(*trans*)-*p*-menthane-3,8-diol; (±)-(*cis*)-*p*-menthane-3,8-diol [43]; 6-[1-(*p*-hydroxyphenyl) ethyl]-7-*O*-methyl aromadendrin [40]; citronellol acetate [44]; 3,5,4′,5″-tetrahydroxy-7-methoxy-6-[1-(*p*-hydroxy-phenyl)ethyl]flavanone; 3,5,7,4′,5″-pentahydroxy-6-[1-(*p*-hydroxy-phenyl)ethyl] flavanone [45]; 1-*O*,2-*O*-digaloil-6-*O*-*trans*-*p*-coumaroyl-β-D-glucoside; 1-*O*-*trans*-*p*-coumaroyl-6-*O*-cinamoil-β-D-glucoside; α- and β-6-*O*-*trans*-*p*-coumaroyl-D-glucoside; 7-methylaromadendrin-4′-*O*-6″-*trans*-*p*-coumaroyl-β-D-glucoside; aromadendrin; aromadendrin-7-methyl ether; naringenin; sakuranetin; kaempferol-7-methyl ether; gallic acid [46]; citriodora A *; 3β,7β,25-trihydroxycucurbita-5,23-(*E*)-dien-19-al; kuguacin A; kuguacin H; 3β,7β-dihydroxy-25-methoxycucurbita-5,23-(*E*)-dien-19-al; kuguacin S [47]; *trans*-calamenene; T-muurolol; α-cadinol; 2β-hydroxy-α-cadinol; 4-hydroxy-3,5-dimethoxybenzaldehyde; 4-hydroxy-3,5-dimethoxybenzoic acid; linoleic acid; squalene; α-tocopherol; erythrodiol; morolic acid; betulonic acid; cycloeucalenol; cycloeucalenol vernolitate *; β-sitosterol; β-sitosteryl-β-D-glucoside; sitostenone; yangambin; sesamin [48]; rhamnocitrin; 6-[1-(*p*-hydroxyphenyl)ethyl]-7-*O*-methyl aromadendrin *; 6-[1-(*p*-hydroxyphenyl)ethyl]-rhamnocitrin; kaempferol; 7-*O*-methyl aromadendrin [49]; citriodolic acids A–C *; rosmarinic acid; ferulic acid; gallic acid [50]; ellagic acid; gallic acid; quercetin; myricetin; 3-*O*-methylellagic acid-4′-*O*-α-L-rhamnoside; quercetin-3-*O*-β-D-galactoside; kaempferol-3-*O*-β-D-glucoside; quercetin-3-*O*-β-D-glucuronide; quercetin-3-*O*-rutinoside; 3,3′,4-tri-*O*-methylellagic acid-4′-*O*-β-D-glucopyranosyl [39]; rhodomyrtosone E *; betulinic acid; oleanolic acid; ursolic acid; corosolic acid; asiatic acid; madasiatic acid; euscaphic acid; 5,7,4′-trihydroxy dihydroflavanol; isoquercitrin; isomyricitrin; myricitrin; gallic acid [51]; citrioside A *; hesperidin; baicalin; puerarin; trifolirhizin 6′-monoacetate; trifolirhizin [52]; citrioside C *; kaempferol-3-*O*-β-D-glucopyranosyl (12)-α-L-rhamnoside; kaempferol-3-*O*-α-L-rhamnoside; quercetin-3-*O*-α-L-rhamnoside [53]; 7-*O*-methylaromadendrin; aromadendrin-dimethyl ether; 7-*O*-methylkempferol; ellagic acid [54].	**Anti-Fungal Activity:** potent fungicidal activity of leaf EO against *C*. *albicans*, *C*. *krusei* and *C*. *tropicalis* [55]; anti-fungal activity of leaf EO against *M. canis*, *M. gypseum*, *T. mentagrophytes* and *T. rubrum* [56]; anti-fungal activity of leaf EO against *A. alternata*, *C. lunata* and *B. specifera* [57]; leaf EO enhanced wound healing rate of *C. albicans*-infected wounds in rats [44]; anti-fungal activity of petroleum ether leaf extract [58]; anti-fungal activity observed in leaf/twig EO [33]; fungicidal activity of leaf EO against *C. albicans* [36]; anti-fungal activity against *P. notatum*, *A. niger* and *F. oxysporium* observed for 7-*O*-methylaromadendrin, 7-*O*-methylkaempferol and ellagic acid [54]. **Anti-Bacterial Activity:** anti-bacterial activity of leaf extracts against *M. aureus*, *E. coli* and *My. Pheli* [54]; anti-bacterial activity of petroleum ether leaf extract [58]; anti-bacterial activity observed from leaf/twig EO [33]; anti-bacterial activity of leaf EO against *S. sanguinis* and *S. salivarius* with anti-biofilm activity [18]; bactericidal activity of leaf EO against *E. coli* and *S. aureus* [36]; bactericidal activity of leaf EO [37]; anti-bacterial activity of aqueous EtOH leaf extract [59]; anti-bacterial activity of fruit and twig EOs against several species [60]; anti-bacterial activity of leaf extract against *S. aureus* [61]; airborne TB inhibition by volatile leaf EO components [62]; leaf EO inhibits the growth of *V. campbellii* BB120 bacteria [63] and treatment of brine shrimp infected with *V. campbellii* with EO enabled their survival [64]. **Acaricidal Activity:** acaricidal activity of leaf EO against *A. nitens* larvae [65]; leaf EO and citronellal reduced *R. microplus* reproductive parameters and increased larval mortality [66]. **Anti-Protozoal Activity:** anti-trypanosomal activity of leaf EO against *T. brucei* [67], *T. evansi* [67] and *T. cruzi* [68]; anti-trypanosomal activity of EtOH extract against *T. brucei* [69]; anti-plasmodial activity observed against *P*. *falciparum* 3D7 and INDO strains [70]. **Anti-Viral Activity:** potent anti-viral activity against RSV observed in citriodolic acids A–C [50], citrioside A [52] and quercetin-3-*O*-α-L-rhamnoside [53]. **Insecticidal Activity:** fumigant activity against the *R. speratus* [71]; larvicidal activity against *A. aegypti* [72]; larvicidal activity of leaf hexane extract against *An. Stephensi*, *Cx. Quinquefasciatus* and *Ae. Aegypti* [73]; larvicidal activity of aqueous EtOH leaf extract [59]; larvicidal activity of leaf EOs against *S. frugiperda* larvae [74]; insecticidal activity of MeOH extract against *T. castaneum* [75]. **Anti-Oxidant/Anti-Inflammatory Activity:** leaf EO showed significant inhibition and IC_50_ values of 4.8–344 µg/mL in DPPH assays [31,33,76,77,78,79,80]; floral EO showed moderate DPPH inhibition [31]; leaf EO showed potent peroxidation inhibition in a linoleic acid/β-carotene assay [33]; leaf and floral EOs showed micromolar protease inhibition [31]; anti-inflammatory properties via inhibition of LOX-1 [28]; kino EtOH extract [32] and flavanols isolated from kino exhibited 15-LOX inhibition [49]; potential anti-inflammatory and anti-viral activity of leaf EO via LOX and ACE2 inhibition [81]; potent anti-inflammatory and gastroprotective properties of ellagitannin fraction in rats [20]; potent inhibition of LPS-induced inflammation in RAW264.7 macrophages [82]; ellagic acid isolated from *C. citriodora* leaves showed anti-inflammatory and gastroprotective activity in an EtOH-induced acute gastric ulcer mouse model [39]; leaf EO showed significant anti-inflammatory and analgesic activity in rats and mice [35]. **Anti-Diabetic Activity:** betulinic acid and corosolic acid isolated from *C. citriodora* leaves enhanced GLUT-4 translocation activity [51]; aqueous leaf extract enhanced insulin secretion and glucose uptake in vitro and had anti-diabetic effects in high-fat-fed rats [80,83]; EtOH leaf extract had anti-diabetic and insulinotropic activity in high-fat-fed rats [21]. **Anti-Cancer Activity:** Anti-proliferative activity of aqueous extract against MIA, PaCa-2, BxPC-3, CFPAC-1 and HPDE cells [30]; leaf EO exhibited anti-proliferative activity against THP-1 cells [84]; EtOAc fraction of EtOH kino extract and isolated 6-[1-(*p*-hydroxyphenyl) ethyl]-7-*O*-methylaromadendrin exhibited potent anti-proliferative activity and apoptosis induction in B16F10 melanoma cells [40]; aqueous fraction of EtOH kino extract inhibited cell growth and induced apoptosis in HepG2 cells [41]; leaf EO showed potent anti-cancer activity against HCT-116, MCF-7 and hepG-2 cells [76]; moderate cytotoxicity of leaf EO against A-549, PC-3, T98G and T47D cells [57]; fruit EO was cytotoxic toward A549, HeLa and CHOK1 cells [85]. **Other Bioactivity:** aqueous extract of leaves and branches detoxified mycotoxins aflatoxins B_1_ and B_2_ [86]; leaf EO exhibited anti-spasmodic effects via inhibition of acetylcholine-induced contraction of a rat ileum [29]; mosquito repellence [43,87]; acetone leaf extract delayed loss of climbing ability and reduced oxidative stress in transgenic *Drosophila* expressing h-αS in the neurons [88].
*C. dichromophlo-ia* (F. Muell.)	Australia	–	Kino infusions are used to treat respiratory complaints [17]; mixed with water as a general tonic and analgesic mouth rinse for toothaches [17,89]; mixed with water for sore eyes, lips, wounds, skin lesions, burns, scabies, cramps and sore throats [17]; kino sucked or decoction prepared as tonic for cardiac complaints [17,89]. Leaves are boiled in water and consumed for respiratory conditions [22].	–	–
*C. eximia* (Schauer)	Australia	L	Dharawal people use leaves to treat colds, fever, chest and muscle pain, extreme diarrhoea and syphilitic sores and as a wash for joints [90].	–	Ethanolic leaf extract showed anti-inflammatory properties in RAW 264.7 macrophages [90].
*C. gummifera* (Gaertn.)	Australia	K, L	Leaves used for respiratory conditions and as a wash for joints [90]. Leaves and kino are used as haemostatics and to treat diarrhoea, ringworm, venereal sores and other STIs [17,91].	Aromadendrin; ellagic acid [24].	Moderate anti-inflammatory activity observed in RAW 264.7 macrophages [90].
*C. intermedia* (R.T. Baker)	Australia	K, L, FL	The Yaegl aboriginal community uses kino to treat warts and wounds and as a haemostatic [92,93].	Intermediones A–D *; (4*S*)-ficifolidione [19].	Intermediones A, B and D showed moderate anti-plasmodial activity against *P. falciparum* 3D7 [19]; intermedianone A also displayed anti-proliferative activity against HEK-293 cells [19].
*C. maculata* (Hook.)	Australia, Egypt, India, Nigeria	L, K, B	Kino is applied directly to burns, and used to treat muscle aches, cramps, wounds, scabies, ringworm, venereal sores, muscle aches and cramps [94]; kino is also ingested to treat coughs, colds, influenza and other infections, dysentery and diarrhoea [94]; kino is also used to treat chronic bowel inflammation [17]. Dharawal people use leaves to treat respiratory infections, fever, chest and muscle pain, and as a wash for joints [90]; juice extracted from the leaves is used to treat paralysis and rheumatism in India [30]. In Australian bush medicine, gum derived from the bark is used to treat bladder infections [30].	β-Germacrenol [95]; 8-demethyl eucalyptin; 8-demethyleucalyptin; myrciaphenone A–B; quercetin-3-*O*-β-D-xyloside; myricetin-3-*O*-α-L-rhamnoside; quercetin-3-*O*-β-D-galactoside; quercetin-3-*O*-β-D-glucoside; quercetin-3-*O*-α-L-rhamnoside; syringic acid; gallic acid-3-methyl ether; gallic acid-4-methyl ether; gallic acid [96]; ellagic acid; *p*-coumaric acid; naringenin; 7-*O*-methylaromadendrin [97]; leucopeargoniidin-3-*O*-α-L-rhamno-β-D-glucoside *; 5,7-dihydroxy 4′-methoxy flavanone [98]; cinnamic acid; 7-*O*-methyl aromadendrin; sakuranetin; 1,6-dicinnamoyl-*O*-α-D-glucoside* [99]; *p*-coumaric acid; 1-*O*-cinnamoyl 6-*O*-coumaroyl-β-D-glucoside *; 7-methylaromadendrin-4′-*O*-(6′′-*trans*-*p*-coumaroyl)-β-D-glucoside * [100]; 3β,13β-dihydroxy-urs-11-en-28-oic acid [101]; 6-[1-(*p*-hydroxyphenyl)ethyl]-7-*O*-methylaromadendrin [40].	Potent anti-leishmanial activity against *L. donovani* observed in eucalyptin, Myciaphenone A and five flavonoid glycosides [96]; potent anti-trypanosomal activity against *T. brucei* [101]; leaf aqueous extract inhibited PaCa-2 cell proliferation [30]; MeOH extract showed anti-inflammatory properties in vitro [102]; EtOH leaf extract exhibited anti-inflammatory properties in RAW 264.7 macrophages [90]; MeOH kino extract showed significant anti-inflammatory properties in rats [102]; 7-*O*-methylaromadendrin, sakuranetin and 1,6-dicinnamoyl-*O*-α-D-glucoside isolated from the kino [99] exhibited anti-oxidant and hepatoprotective properties in rats [103].
*C. opaca* (D.J. Carr & S.G.M. Carr)	Australia	–	Kino is applied directly to scabies, cuts and sores, and the gum is boiled in water and applied to sore eyes [22].	–	–
*C. papuana* (F. Muell.)	Australia	B	Kino is used as a decoction for sores, cramps, burns, pains and cuts, skin lesions, scabies, sore throat and cough; infusions are used for colds and sore eyes [17].	Morolic acid [104].	–
*C. polycarpa* (F. Muell.)	Iran	L	Kino is used to treat sores, burns, cuts, burns, yaws, ulcers, dysentery and toothaches [17,91].	– † [105,106]	Anti-bacterial activity of leaf EO against *S. aureus* [107].
*C. terminalis* (F. Muell.)	Australia	–	Kino is applied to wounds, cuts, sores, toothaches, scabies, skin lesions scabies and cramps [17,22]; it is also taken in water for diarrhoea, headaches, coughs, heart disease and blood conditions [17,22,89]. Bark is used to treat dysentery [91].	– ‡ [108]	–
*C. tessellaris* (F. Muell.)	Australia	–	Kino is consumed for dysentery [17]; gum is used for constipation [91].	–† [109]	–
*C. torelliana* (F. Muell.)	Australia, Papua New Guinea, Nigeria	K, L, B FR, FL	Leaves are used to treat gastrointestinal disorders, sore throats, bacterial respiratory and urinary tract infections [110]; leaf poultice is applied to ulcers and wounds [110]; hot water extracts of leaves are used in Nigerian traditional medicine as an analgesic, anti-inflammatory, cancer treatment and to alleviate intestinal disorders [111].	Torellianones A–F *; torellianol A *; ficifolidione; (4*R*)-ficifolidione; kunzeanone A–B [112]; (+)-pinene; (±)-α-pinene; (-)-β-pinene; ocimene; (+)-aromadendrene; benzaldehyde [113]; 5-hydroxy-7,4′-dimethoxy-6-methylflavone [114]; hydroxymyristic acid methyl ester; methyl (*E*)- and (*Z*)-6-(8-oxooctadecahydrochrysen-1-yl)non-7-enoate [115]; (2*S*)-cryptostrobin; (2*S*)-stroboponin; (2*S*)- cryptostrobin 7-methyl ether; (2*S*)- desmethoxymatteucinol; (2*S*)-pinostrobin; (2*S*)-pinocembrin [116]; 3,4′,5,7-tetrahydroxyflavanone; 3′,4′,5,7-tetrahydroxyflavanone; 4′,5,7-trihydroxyflavanone; 3,4′,5-trihydroxy-7-methoxyflavanone; (+)-(2S)-4′,5,7-trihydroxy-6-methylflavanone; 4′,5,7-trihydroxy-6,8-dimethylflavanone; 4′,5-dihydroxy-7-methoxyflavanone [117].	Torellianones C–F, (4*R*)- and (4*S*)-ficifolidones and kunzeanone A exhibited anti-plasmodial activity against *P. falciparum* [112]; potent in vitro anti-*H. pylori* activity of leaf and stem extracts across various strains [110]; leaf and stem bark extracts and isolated compounds showed anti-TB activity [115]; anti-bacterial activity of stingless bee propolis, fruit resin and isolated flavonoids against *S. aureus* [116]; moderate anti-bacterial activities and potent cytotoxicity to PC-3, Hep G2, Hs 578T and MDA-MB-231 exhibited by leaf and fruit EOs [111]; anti-tuberculosis activity observed in hydroxymyristic acid methyl ester and methyl (*E*)- and (*Z*)-6-(8-oxooctadecahydrochrysen-1-yl)non-7-enoate [115]; MeOH extract of leaves and bark showed anti-secretory and gastroprotective properties in rats with EtOH/HCl-induced ulceration [118].

^§^ Code for part of plant studied: L = leaves; K = kino/resin/exudate; B = bark; FR = fruit; FL = flowers; T = twigs; H = heartwood. * Novel compound(s) that were isolated. † No compounds were isolated; however, a chemical profile of the leaf EO was reported. ‡ Raman spectrum of kino previously obtained.

**Table 2:** The two major constituents of the leaf EO of selected *Corymbia* species, expressed as percentages of total EO content. The *Corymbia* spp. are abbreviated as follows: *Ble.*—*bleeseri*; *Cal.—calophylla*; *Cit.*—*citriodora*; *Exi.*—*eximia*; *Gum.*—*gummifera*; *Int.*—*intermedia*; *Mac.*—*maculata*; *Pol.*—*polycarpa*; *Tes.*—*tessellaris* and *Tor.*—*torelliana*.

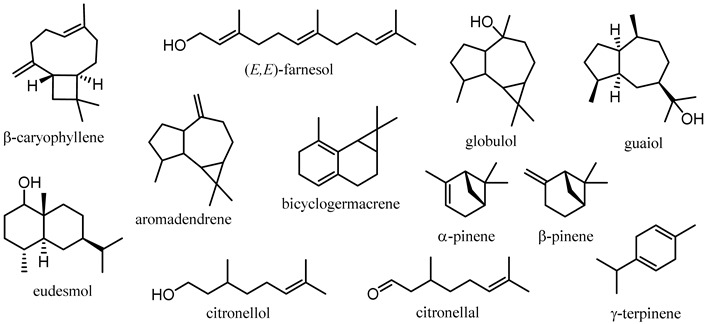										
	***Corymbia*** **spp.**									
	***Ble.*** **[23]**	***Cal.*** **[155]**	***Cit.*** **[106]**	***Exi.*** **[109]**	***Gum.*** **[23]**	***Int.*** **[150]**	***Mac.*** **[150]**	***Pol.*** **[105]**	***Tes.*** **[23]**	***Tor.*** **[151]**
aromadendrene	-	-	-	-	-	-	-	-	16.0	-
bicyclogermacrene	33.2	-	-	-	34.3	-	-	-	-	-
β-caryophyllene	5.2	-	-	-	6.8	-	-	-	-	7.4
citronellal	-	-	66.0	-	-	-	-	-	-	-
citronellol	-	-	12.1	-	-	-	-	-	-	-
α-eudesmol	-	-	-	17.7	-	-	-	-	-	-
(*E*,*E*)-farnesol	-	21.3	-	-	-	-	-	-	-	-
globulol	-	-	-	-	-	-	-	-	5.3	-
guaiol	-	-	-	-	-	-	8.8	-	-	-
α-pinene	-	-	-	33.4	-	18.5	68.1	22.4	-	69.6
β-pinene	-	-	-	-	-	24.6	-	41.5	-	-
γ-terpinene	-	12.1	-	-	-	-	-	-	-	-

**Table 3:** Major constituents of the EOs isolated from the leaf, twig, fruit and/or flowers in *C. citriodora* and *C. torelliana*. Values are expressed as percentages of total EO content.

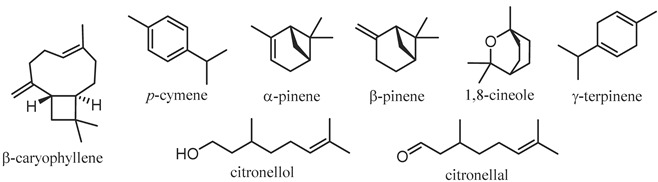						
	***C. citriodora***				***C. torelliana***	
	**Leaf [106]**	**Twig [60]**	**Fruit [85]**	**Flower [142]**	**Leaf [151]**	**Flower [111]**
β-caryophyllene	-	-	-	-	7.4	-
1,8-cineole	-	17.7	-	-	-	-
citronellal	66.0	-	-	-	-	-
citronellol	12.1	-	-	9.9	-	-
*p*-cymene	-	17.1	-	-	-	-
α-pinene	-	-	54.1	16.4	69.6	55.8
β-pinene	-	-	-	-	-	10.8
γ-terpinene	-	-	8.6	-	-	-

**Table 4:** Summary of major phytochemical classes previously isolated from *Corymbia* species. The *Corymbia* spp. are abbreviated as follows: *Cal.*—*calophylla*; *Cit.*—*citriodora*; *Gum.*—*gummifera*; *Int.*—*intermedia*; *Mac.*—*maculata*; *Pap.*—*papuana* and *Tor.*—*torelliana*.

	*Corymbia* spp.							
	*Cal.*	*Cit.*	*Gum.*	*Int.*	*Mac.*	*Pap.*	*Tor.*	Total *
**Alkaloids**	**-**	**-**	**-**	**-**	**-**	**-**	**-**	**-**
**Polyketides**	**-**	**4**	**-**	**5**	**-**	**-**	**11**	**20**
Simple	-	3	-	-	-	-	-	3
β-Triketones	-	1	-	5	-	-	11	17
**Terpenoids**	**1**	**28**	**-**	**-**	**2**	**1**	**6**	**37**
Monoterpenoids	-	3	-	-	-	-	4	7
Sesquiterpenoids	-	4	-	-	1	-	1	6
Diterpenoids	-	-	-	-	-	-	-	-
Sesterpenoids	-	-	-	-	-	-	1	1
Triterpenoids	1	21	-	-	1	1	-	23
**Polyphenols**	**8**	**52**	**2**	**-**	**24**	**-**	**15**	**88**
Flavonoids	6	35	1	-	12	-	14	60
Phenolic acids	2	15	1	-	12	-	1	26
Lignans	-	2	-	-	-	-	-	2
**Fatty Acids**	**-**	**1**	**-**	**-**	**-**	**-**	**1**	**2**
**Total**	**9**	**85**	**2**	**5**	**27**	**1**	**33**	**147**

* Note: totals are adjusted to account for compounds isolated in multiple *Corymbia* spp.

## Data Availability

No data were used for the research described in the article.

## Abbreviations

DPPH	1,1-diphenyl-2-picrylhydrazyl
ACE	acetylcholine-converting enzyme
COX	cyclooxygenase
EO	essential oil
EtOH	ethanol
EtOAc	ethyl acetate
GLUT	glucose transporter
h-αS	human alpha synuclein
iNOS	inducible nitric oxide synthase
IL	interleukin
LOX	lipoxygenase
LPS	lipopolysaccharide
M^Pro^	main protease
MeOH	methanol
MAPK	mitogen-activated protein kinase
NO	nitric oxide
NF-κB	nuclear factor-kappa B
PGE	prostaglandin
RSV	respiratory syncytial virus
SAR	structure–activity relationship
TNF-α	tumour necrosis factor-α

## References

[1] PhRMA (2020). PhRMA Annual Membership Survey. https://phrma.org/-/media/Project/PhRMA/PhRMA-Org/PhRMA-Org/PDF/P-R/PhRMA_Membership_Survey_2020.pdf.

[2] Dadgostar P. (2019). Antimicrobial Resistance: Implications and Costs. Infect. Drug Resist..

[3] Ford E.S., Giles W.H., Mokdad A.H. (2004). Increasing Prevalence of the Metabolic Syndrome Among US Adults. Diabetes Care.

[4] Conrad N., Misra S., Verbakel J.Y., Verbeke G., Molenberghs G., Taylor P.N., Mason J., Sattar N., McMurray J.J., McInnes I.B. (2023). Incidence, Prevalence, and Co-Occurrence of Autoimmune Disorders Over Time and by Age, Sex, and Socioeconomic Status: A Population-Based Cohort Study of 22 million Individuals in the UK. Lancet.

[5] Li L., Xu H., Qu L., Nisar M., Farrukh Nisar M., Liu X., Xu K. (2023). Water extracts of Polygonum Multiflorum Thunb. and its active component emodin relieves osteoarthritis by regulating cholesterol metabolism and suppressing chondrocyte inflammation. Acupunct. Herb. Med..

[6] Abidullah S., Rauf A., Zaman W., Ullah F., Ayaz A., Batool F., Saqib S. (2023). Consumption of wild food plants among tribal communities of Pak-Afghan border, near Bajaur, Pakistan. Acta Ecol. Sin..

[7] Calixto J.B. (2005). Twenty-Five Years of Research on Medicinal Plants in Latin America: A Personal View. J. Ethnopharmacol..

[8] Zaman W., Ye J., Saqib S., Liu Y., Shan Z., Hao D., Chen Z., Xiao P. (2021). Predicting potential medicinal plants with phylogenetic topology: Inspiration from the research of traditional Chinese medicine. J. Ethnopharmacol..

[9] Newman D.J., Cragg G.M. (2007). Natural Products as Sources of New Drugs Over the Last 25 Years. J. Nat. Prod..

[10] Schippmann U., Leaman D.J., Cunningham A.B. (2002). Impact of Cultivation and Gathering of Medicinal Plants on Biodiversity: Global Trends and Issues. Biodiversity and the Ecosystem Approach in Agriculture, Forestry and Fisheries.

[11] Kesharwani V., Gupta S., Kushwaha N., Kesharwani R., Patel D.K. (2018). A review on therapeutics application of eucalyptus oil. Int. J. Herb. Med..

[12] Turnbull J. (1981). Eucalypts in China. Aust. For..

[13] Silva-Pando F., Pino-Pérez R. (2016). Introduction of *Eucalyptus* into Europe. Aust. For..

[14] Vuong Q.V., Chalmers A.C., Jyoti Bhuyan D., Bowyer M.C., Scarlett C.J. (2015). Botanical, phytochemical, and anticancer properties of the *Eucalyptus* species. Chem. Biodivers..

[15] Rozefelds A. (1996). *Eucalyptus* Phylogeny and History: A Brief Summary. Tasforests.

[16] Hill K.D., Johnson L.A. (1995). Systematic Studies in the Eucalypts 7. A Revision of the Bloodwoods, genus *Corymbia* (Myrtaceae). Telopea.

[17] Locher C., Currie L. (2010). Revisiting Kinos—An Australian Perspective. J. Ethnopharmacol..

[18] Marinkovic J., Markovic T., Nikolic B., Soldatovic I., Ivanov M., Ciric A., Sokovic M., Markovic D. (2021). Antibacterial and Antibiofilm Potential of *Leptospermum petersonii* F.M.Bailey, *Eucalyptus citriodora* Hook. *Pelargonium graveolens* L′Hér. and *Pelargonium roseum* (Andrews) DC. Essential Oils Against Selected Dental Isolates. J. Essent. Oil-Bear. Plants.

[19] Senadeera S.P.D., Robertson L.P., Duffy S., Wang Y., Avery V.M., Carroll A.R. (2018). β-Triketone-Monoterpene Hybrids from the Flowers of the Australian Tree *Corymbia intermedia*. J. Nat. Prod..

[20] Al-Sayed E., El-Naga R.N. (2015). Protective Role of Ellagitannins from *Eucalyptus citriodora* Against Ethanol-Induced Gastric Ulcer in Rats: Impact on Oxidative Stress, Inflammation and Calcitonin-Gene Related Peptide. Phytomedicine.

[21] Ansari P., Choudhury S.T., Abdel-Wahab Y.H.A. (2022). Insulin Secretory Actions of Ethanol Extract of *Eucalyptus citriodora* Leaf, Including Plasma DPP-IV and GLP-1 Levels in High-Fat-Fed Rats, as Well as Characterization of Biologically Effective Phytoconstituents. Metabolites.

[22] Smith N.M. (1991). Ethnobotanical Field Notes from The Northern Territory, Australia. J. Adel. Bot. Gard..

[23] Bignell C., Dunlop P., Brophy J. (1997). Volatile Leaf Oils of Some Queensland and Northern Australian Species of the Genus *Eucalyptus* (series II). Part II. Subgenera (a) Blakella, (b) Corymbia, (c) Unnamed, (d) Idiogenes, (e) Monocalyptus and (f) Symphyomyrtus. Flavour Fragr. J..

[24] Hills W.E. (1952). The Chemistry of Eucalypt Kinos. II. Aromadendrin, Kaempferol, and Ellagic acid. Aust. J. Chem..

[25] White D.E., Zampatti L.S. (1952). The Chemistry of Western Australian Plants. Part VII.* Oleanolic Acid Acetate from *Eucalyptus calophylla* Bark. J. Chem. Soc..

[26] Hillis W.E., Carle A. (1960). The Chemistry of Eucalypt Kinos. III. (+)-Afzelechin, Pyrogallol, and (+)-Catechin from *Eucalyptus calophylla* Kino. Aust. J. Chem..

[27] Ganguly A.K., Seshadri T.R. (1961). 543. Leucoanthocyanidins of Plants. Part III. Leucopelargonidin from *Eucalyptus calophylla* Kino. J. Chem. Soc..

[28] Bedi Sahouo G., Tonzibo Z.F., Boti B., Chopard C., Mahy J.P., N’Guessan Y.T. (2003). Anti-Inflammatory and Analgesic Activities: Chemical Constituents of Essential Oils of *Ocimum gratissimum*, *Eucalyptus citriodora* and *Cymbopogon giganteus* Inhibited Lipoxygenase L-1 and Cyclooxygenase of PGHS. Bull. Chem. Soc. Ethiop..

[29] Ayinde B.A., Owolabi O.J. (2012). Inhibitory Effects of the Volatile Oils of *Callistemon citrinus* (Curtis) Skeels and *Eucalyptus citriodora* Hook (Myrtaceae) on the Acetylcholine Induced Contraction of Isolated Rat Ileum. Pak. J. Pharm. Sci..

[30] Bhuyan D.J., Vuong Q.V., Bond D.R., Chalmers A.C., van Altena I.A., Bowyer M.C., Scarlett C.J. (2017). Exploring the Least Studied Australian Eucalypt Genera: *Corymbia* and *Angophora* for Phytochemicals with Anticancer Activity Against Pancreatic Malignancies. Chem. Biodivers..

[31] Gopan R., Renju K.V., Pradeep N.S., Sabulal B. (2009). Volatile Oil of *Eucalyptus citriodora* Hook, from South India: Chemistry and Antibacterial Activity. Int. J. Essent. Oil Ther..

[32] Hung W.J., Chen Z.T., Lee S.W. (2018). Antioxidant and Lipoxygenase Inhibitory Activity of the Kino of *Eucalyptus citriodora*. Indian J. Pharm. Sci..

[33] Salem M.Z.M., Elansary H.O., Ali H.M., El-Settawy A.A., Elshikh M.S., Abdel-Salam E.M., Skalicka-Woźniak K. (2018). Bioactivity of Essential Oils Extracted from *Cupressus macrocarpa* Branchlets and *Corymbia citriodora* Leaves Grown in Egypt. BMC Complement Altern. Med..

[34] Ribeiro R.V., Bieski I.G.C., Balogun S.O., Martins D.T.D.O. (2017). Ethnobotanical study of Medicinal Plants Used by Ribeirinhos in the North Araguaia Microregion, Mato Grosso, Brazil. J. Ethnopharmacol..

[35] Silva J., Abebe W., Sousa S.M., Duarte V.G., Machado M.I.L., Matos F.J.A. (2003). Analgesic and Anti-Inflammatory Effects of Essential Oils of *Eucalyptus*. J. Ethnopharmacol..

[36] Koudoro Y.A., Agbangnan Dossa C.P., Yèhouénou B.B., Tchobo F.P., Alitonou G.A., Avlessi F., Sohounhloué D.C.K. (2014). Phytochemistry, Antimicrobial and Antiradical Activities Evaluation of Essential Oils, Ethanolic and Hydroethanolic Extracts of the Leaves of *Eucalyptus citriodora* Hook. from Benin. Sci. St. Res. Chem. Chem. Eng. Biotechnol. Food Ind..

[37] Mishra M.P., Padhy R.N. (2013). In vitro Antibacterial Efficacy of 21 Indian Timber-Yielding Plants Against Multidrug-Resistant Bacteria Causing Urinary Tract Infection. Osong Public Health Res. Perspect..

[38] Akin-Osanaiye B.C., Agbaji A.S., Dakare M.A. (2007). Antimicrobial Activity of Oils and Extracts of *Cymbopogon citratus* (Lemon Grass), *Eucalyptus citriodora* and *Eucalyptus camaldulensis*. J. Med. Sci..

[39] Yu Q., Feng Z., Huang L., He J., Zhou Z., Liu F. (2021). Ellagic Acid (EA), a Tannin was Isolated from *Eucalyptus citriodora* Leaves and its Anti-Inflammatory Activity. Med. Chem. Res..

[40] Duh P.D., Chen Z.T., Lee S.W., Lin T.P., Wang Y.T., Yen W.J., Kuo L.F., Chu H.L. (2012). Antiproliferative Activity and Apoptosis Induction of *Eucalyptus citriodora* Resin and its Major Bioactive Compound in Melanoma B16F10 Cells. J. Agric. Food Chem..

[41] Shen K.H., Chen Z.T., Duh P.D. (2012). Cytotoxic Effect of *Eucalyptus citriodora* Resin on Human Hepatoma HepG2 Cells. Am. J. Chin. Med..

[42] Anet E.F.L.J., Birch A.J., Massy-Westropp R.A. (1957). The Isolation of Shikimic Acid from *Eucalyptus citriodora* Hook. Aust. J. Chem..

[43] Barasa S.S., Ndiege I.O., Lwande W., Hassanali A. (2002). Repellent Activities of Stereoisomers of *p*-Menthane-3,8-diols Against *Anopheles gambiae* (Diptera: Culicidae). J. Med. Entomol..

[44] El-Sakhawy M.A., Soliman G.A., El-Sheikh H.H., Ganaie M.A. (2023). Anticandidal Effect of *Eucalyptus* Oil and Three Isolated Compounds on Cutaneous Wound Healing in Rats. Eur. Rev. Med. Pharmacol..

[45] Freitas M.O., Lima M.A.S., Silveira E.R. (2007). NMR Assignments of Unusual Flavonoids from the Kino of *Eucalyptus citriodora*. Magn. Reson. Chem..

[46] Freitas M.O., Lima M.A.S., Silveira E.R. (2007). Polyphenol Compounds of the Kino of *Eucalyptus citriodora*. Quim. Nova.

[47] He Y., Shang Q., Tian L. (2015). A New Triterpenoid and Potential Anticancer Cytotoxic Activity of Isolated Compounds from the Roots of *Eucalyptus citriodora*. J. Chem. Res..

[48] Lee C.K., Chang M.H. (2000). The Chemical Constituents from the Heartwood of *Eucalyptus citriodora*. J. Chin. Chem. Soc..

[49] Lee S.W., Hung W.J., Chen Z.T. (2017). A New Flavonol from the Kino of *Eucalyptus citriodora*. Nat. Prod. Res..

[50] Lin S.Q., Zhou Z.L., Yin W.Q. (2016). Three New Polyphenolic Acids from the Leaves of *Eucalyptus citriodora* with Antivirus Activity. Chem. Pharm. Bull..

[51] Wang C., Yang J., Zhao P., Zhou Q., Mei Z., Yang G., Yang X., Feng Y. (2014). Chemical Constituents from *Eucalyptus citriodora* Hook Leaves and their Glucose Transporter 4 Translocation Activities. Bioorg. Med. Chem. Lett..

[52] Zou X., Huang D., Zhou C., Li L., Chen K., Guo Z., Lin S., Yin W., Zhou Z. (2014). A New Flavonoid Glycoside from the Leaves of *Eucalyptus citriodora*. J. Chem. Res..

[53] Zhou Z.L., Yin W.Q., Zou X.P., Huang D.Y., Zhou C.L., Li L.M., Chen K.C., Guo Z.Y., Lin S.Q. (2014). Flavonoid Glycosides and Potential Antivirus Activity of Isolated Compounds from the Leaves of *Eucalyptus citriodora*. J. Korean Soc. Appl. Biol. Chem..

[54] Satwalekar S., Gupta T., Narasimharao P. (1957). Chemical and Antibacterial Properties of Kino from *Eucalyptus* spp. Citriodorol—The Antibiotic Principle from the Kino of *E. Citriodora*. J. Indian Inst. Sci..

[55] Cavalcanti A.L., Aguiar Y.P.C., Santos F.G.D., Cavalcanti A.F.C., De Castro R.D. (2017). Susceptibility of *Candida albicans* and *Candida* non-*albicans* Strains to Essential Oils. Biomed. Pharmacol. J..

[56] Tolba H., Moghrani H., Benelmouffok A., Kellou D., Maachi R. (2015). Essential Oil of Algerian *Eucalyptus citriodora*: Chemical Composition, Antifungal Activity. J. Mycol. Med..

[57] Gupta S., Bhagat M., Sudan R., Dogra S., Jamwal R. (2015). Comparative Chemoprofiling and Biological Potential of Three *Eucalyptus* Species Growing in Jammu and Kashmir. J. Essent. Oil-Bear. Plants.

[58] Sharma A., Sharma K. (2011). Efficacy of *Lawsonia inermis* Linn. and *Eucalyptus citriodora* Hook. Extracts as Inhibitors of Growth and Enzymatic Activity of *Aspergillus flavus* and *A. parasiticus*. J. Biol. Act. Prod. Nat..

[59] Udaya Prakash N.K., Bhuvaneswari S., Sripriya N., Arulmozhi R., Kavitha K., Aravitha R., Bharathiraja B. (2014). Studies on Phytochemistry, Antioxidant, Antibacterial, Larvicidal and Pesticidal Activities of Aromatic Plants from Yelagiri Hills. Int. J. Pharm. Pharm. Sci..

[60] Su Y.C., Hsu K.P., Ho C.L. (2017). Composition, in vitro Antibacterial and Anti-Mildew Fungal Activities of Essential Oils from Twig and Fruit Parts of *Eucalyptus citriodora*. Nat. Prod. Commun..

[61] Da Cruz J.E.R., Da Costa Guerra J.F., De Souza Gomes M., Freitas G.R.O., Morais E.R. (2019). Phytochemical Analysis and Evaluation of Antimicrobial Activity of *Peumus boldus*, *Psidium guajava*, *Vernonia polysphaera*, *Persea americana*, *Eucalyptus citriodora* Leaf Extracts and *Jatropha multifida* Raw Sap. Curr. Pharm. Biotechnol..

[62] Ramos Alvarenga R.F., Wan B., Inui T., Franzblau S.G., Pauli G.F., Jaki B.U. (2014). Airborne Antituberculosis Activity of *Eucalyptus citriodora* Essential Oil. J. Nat. Prod..

[63] Zheng X., Feyaerts A.F., Dijck P.V., Bossier P. (2020). Inhibitory Activity of Essential Oils Against *Vibrio campbellii* and *Vibrio parahaemolyticus*. Microorganisms.

[64] Zheng X., Han B., Kumar V., Feyaerts A.F., Van Dijck P., Bossier P. (2021). Essential Oils Improve the Survival of Gnotobiotic Brine Shrimp (*Artemia franciscana*) Challenged with *Vibrio campbellii*. Front. Immunol..

[65] Clemente M.A., De Oliveira Monteiro C.M., Scoralik M.G., Gomes F.T., De Azevedo Prata M.C., Daemon E. (2010). Acaricidal Activity of the Essential Oils from *Eucalyptus citriodora* and *Cymbopogon nardus* on Larvae of *Amblyomma cajennense* (Acari: Ixodidae) and *Anocentor nitens* (Acari: Ixodidae). Parasitol. Res..

[66] Rodrigues L., Giglioti R., Gomes A.C.P., Katiki L.M., Otsuk I.P., da Silva Matos R., Nodari E.F., Veríssimo C.J. (2020). In vitro Effect of Volatile Substances from *Eucalyptus* Oils on *Rhipicephalus microplus*. Rev. Bras. Farmacogn..

[67] Habila N., Agbaji A.S., Ladan Z., Bello I.A., Haruna E., Dakare M.A., Atolagbe T.O. (2010). Evaluation of *in vitro* Activity of Essential Oils Against *Trypanosoma brucei brucei* and *Trypanosoma evansi*. J. Parasitol. Res..

[68] Silva Maiolini T.C., Rosa W., de Oliveira Miranda D., Costa-Silva T.A., Tempone A.G., Pires Bueno P.C., Ferreira Dias D., Aparecida Chagas de Paula D., Sartorelli P., Lago J.H.G. (2022). Essential Oils from Different Myrtaceae Species from Brazilian Atlantic Forest Biome–Chemical Dereplication and Evaluation of Antitrypanosomal Activity. Chem. Biodivers..

[69] Jain S., Jacob M., Walker L., Tekwani B. (2016). Screening North American Plant Extracts in vitro Against *Trypanosoma brucei* for Discovery of New Antitrypanosomal Drug Leads. BMC Complement Altern. Med..

[70] Singh N., Kaushik N.K., Mohanakrishnan D., Tiwari S.K., Sahal D. (2015). Antiplasmodial Activity of Medicinal Plants from Chhotanagpur Plateau, Jharkhand, India. J. Ethnopharmacol..

[71] Park I.L.K., Shin S.C. (2005). Fumigant Activity of Plant Essential Oils and Components from Garlic (*Allium sativum*) and Clove Bud (*Eugenia caryophyllata*) Oils Against the Japanese Termite (*Reticulitermes speratus* Kolbe). J. Agric. Food Chem..

[72] Vera S.S., Zambrano D.F., Méndez-Sanchez S.C., Rodríguez-Sanabria F., Stashenko E.E., Duque Luna J.E. (2014). Essential Oils with Insecticidal Activity against Larvae of *Aedes aegypti* (Diptera: Culicidae). Parasitol. Res..

[73] Singh R.K., Dhiman R.C., Mittal P.K. (2007). Studies on Mosquito Larvicidal Properties of *Eucalyptus citriodora* Hook (Family-Myrtaceae). J. Commun. Dis..

[74] Cruz G.S., Wanderley-Teixeira V., da Silva L.M., Dutra K.A., Guedes C.A., de Oliveira J.V., Navarro D.M.A.F., Araújo B.C., Teixeira Á.A.C. (2017). Chemical Composition and Insecticidal Activity of the Essential Oils of *Foeniculum vulgare* Mill. *Ocimum basilicum* L. *Eucalyptus staigeriana* F. Muell. ex Bailey, *Eucalyptus citriodora* Hook and *Ocimum gratissimum* L. and Their Major Components on *Spodoptera frugiperda* (Lepidoptera: Noctuidae). J. Essent. Oil-Bear. Plants.

[75] Sahi N.M. (2016). Evaluation of Insecticidal Activity of Bioactive Compounds from *Eucalyptus citriodora* Against *Tribolium castaneum*. Int. J. Pharmacogn. Phytochem. Res..

[76] Ghareeb M.A., Refahy L.A., Saad A.M., Ahmed W.S. (2016). Chemical Composition, Antioxidant and Anticancer Activities of the Essential Oil from *Eucalyptus citriodora* (Hook.) Leaves. Der Pharma Chem..

[77] Chahomchuen T., Insuan O., Insuan W. (2020). Chemical Profile of Leaf Essential Oils from Four *Eucalyptus* Species from Thailand and Their Biological Activities. Microchem. J..

[78] Jirovetz L., Bail S., Buchbauer G., Stoilova I., Krastanov A., Stoyanova A., Stanchev V., Schmidt E. (2007). Chemical Composition, Olfactory Evaluation and Antioxidant Effects of the Leaf Essential Oil of *Corymbia citriodora* (Hook) from China. Nat. Prod. Commun..

[79] Zhao Q., Bowles E.J., Zhang H.Y. (2008). Antioxidant Activities of Eleven Australian Essential Oils. Nat. Prod. Commun..

[80] Arjun P., Shivesh J., Alakh N.S. (2009). Antidiabetic Activity of Aqueous Extract of *Eucalyptus citriodora* Hook. in Alloxan Induced Diabetic Rats. Pharmacogn. Mag..

[81] Ak Sakallı E., Teralı K., Karadağ A.E., Biltekin S.N., Koşar M., Demirci B., Hüsnü Can Başer K., Demirci F. (2022). In vitro and in silico Evaluation of ACE2 and LOX Inhibitory Activity of *Eucalyptus* Essential Oils, 1,8-Cineole, and Citronellal. Nat. Prod. Commun..

[82] Ho C.L., Li L.H., Weng Y.C., Hua K.F., Ju T.C. (2020). *Eucalyptus* Essential Oils Inhibit the Lipopolysaccharide-Induced Inflammatory Response in RAW264.7 Macrophages Through Reducing MAPK and NF-κB Pathways. BMC Complement. Med. Ther..

[83] Ansari P., Flatt P.R., Harriott P., Abdel-Wahab Y.H.A. (2021). Insulinotropic and Antidiabetic Properties of *Eucalyptus citriodora* Leaves and Isolation of Bioactive Phytomolecules. J. Pharm. Pharmacol..

[84] Miguel M.G., Gago C., Antunes M.D., Lagoas S., Faleiro M.L., Megías C., Cortés-Giraldo I., Vioque J., Figueiredo A.C. (2018). Antibacterial, Antioxidant, and Antiproliferative Activities of *Corymbia citriodora* and the Essential Oils of Eight *Eucalyptus* Species. Medicines.

[85] Koundal R., Kumar D., Walia M., Kumar A., Thakur S., Chand G., Padwad Y.S., Agnihotri V.K. (2016). Chemical and in vitro Cytotoxicity Evaluation of Essential Oil from *Eucalyptus citriodora* Fruits Growing in the Northwestern Himalaya, India. Flavour Fragr. J..

[86] Iram W., Anjum T., Iqbal M., Ghaffar A., Abbas M. (2015). Mass Spectrometric Identification and Toxicity Assessment of Degraded Products of Aflatoxin B1 and B2 by *Corymbia citriodora* Aqueous Extracts. Sci. Rep..

[87] Hadis M., Lulu M., Mekonnen Y., Asfaw T. (2003). Field Trials on the Repellent Activity of Four Plant Products Against Mainly *Mansonia* Population in Western Ethiopia. Phytother. Res..

[88] Siddique Y.H., Mujtaba S.F., Jyoti S., Naz F. (2013). GC-MS Analysis of *Eucalyptus citriodora* Leaf Extract and its Role on the Dietary Supplementation in Transgenic *Drosophila* Model of Parkinson’s Disease. Food Chem. Toxicol..

[89] Reid E.J., Betts T. (1979). Records of Western Australian Plants Used by Aboriginals as Medicinal Agents. Planta Med..

[90] Akhtar M.A., Raju R., Beattie K.D., Bodkin F., Münch G. (2016). Medicinal Plants of the Australian Aboriginal Dharawal People Exhibiting Anti-Inflammatory Activity. J. Evid. Based Complement. Altern. Med..

[91] Turpin G., Ritmejerytė E., Jamie J., Crayn D., Wangchuk P. (2022). Aboriginal Medicinal Plants of Queensland: Ethnopharmacological Uses, Species Diversity, and Biodiscovery Pathways. J. Ethnobiol. Ethnomed..

[92] Packer J., Brouwer N., Harrington D., Gaikwad J., Heron R., Yaegl Community E., Ranganathan S., Vemulpad S., Jamie J. (2012). An Ethnobotanical Study of Medicinal Plants Used by the Yaegl Aboriginal Community in Northern New South Wales, Australia. J. Ethnopharmacol..

[93] Packer J., Naz T., Harrington D., Jamie J.F., Vemulpad S.R. (2015). Antimicrobial Activity of Customary Medicinal Plants of the Yaegl Aboriginal community of Northern New South Wales, Australia: A Preliminary Study. BMC Res. Notes.

[94] von Martius S., Hammer K.A., Locher C. (2012). Chemical Characteristics and Antimicrobial Effects of Some *Eucalyptus* Kinos. J. Ethnopharmacol..

[95] Cornwell C.P., Reddy N., Leach D.N., Grant Wyllie S. (2001). Germacradienols in the Essential Oils of the Myrtaceae. Flavour Fragr. J..

[96] Sidana J., Neeradi D., Choudhary A., Singh S., Foley W.J., Singh I.P. (2013). Antileishmanial Polyphenols from *Corymbia maculata*. J. Chem. Sci..

[97] Gell R.J., Pinhey J.T., Ritchie E. (1958). The Constituents of the Kino of *Eucalyptus maculata* Hook. Aust. J. Chem..

[98] Mishra C.S., Misra K. (1980). Chemical Examination of the Stem Bark of *Eucalyptus maculata*. Planta Med..

[99] Abdel-Sattar E., Kohiel M.A., Shihata I.A., El-Askary H. (2000). Phenolic Compounds from *Eucalyptus maculata*. Pharmazie.

[100] Rashwan O.A. (2002). New Phenylpropanoid Glucosides from *Eucalyptus maculata*. Molecules.

[101] Ebiloma G.U., Igoli J.O., Katsoulis E., Donachie A.M., Eze A., Gray A.I., de Koning H.P. (2017). Bioassay-Guided Isolation of Active Principles from Nigerian Medicinal Plants Identifies New Trypanocides with Low Toxicity and No Cross-Resistance to Diamidines and Arsenicals. J. Ethnopharmacol..

[102] Ali D.E., Abdelrahman R.S., El Gedaily R.A., Ezzat S.M., Meselhy M.R., Abdelsattar E. (2020). Evaluation of the Anti-Inflammatory and Antioxidant Activities of Selected Resin Exudates. Trop. J. Nat. Prod. Res..

[103] Mohamed A.F., Ali Hasan A.G., Hamamy M.I., Abdel-Sattar E. (2005). Antioxidant and Hepatoprotective Effects of *Eucalyptus maculata*. Med. Sci. Monit..

[104] Hart N., Lamberton J. (1965). Morolic acid (3-Hydroxyolean-18-en-28-oic acid) From the Bark of *Eucalyptus papuana* F. Muell. Aust. J. Chem..

[105] Silou T., Loumouamou A.N., Loukakou E., Chalchat J.C., Figuérédo G. (2009). Intra and Interspecific Variations of Yield and Chemical Composition of Essential Oils from Five *Eucalyptus* Species Growing in the Congo-Brazzaville. Corymbia subgenus. J. Essent. Oil Res..

[106] Silou T., Loumouamou A.N., Makany A.R., Dembi F., Figuérédo G., Chalchat J.-C. (2010). Multivariate Statistical Analysis of the Variability of Essential Oils from the Leaves of *Eucalyptus torelliana* Acclimatised in Congo-Brazzaville. J. Essent. Oil-Bear. Plants.

[107] Panahi Y., Sattari M., Babaie A.P., Beiraghdar F., Ranjbar R., Bigdeli M. (2011). The Essential Oils Activity of *Eucalyptus polycarpa*, *E. largiflorence*, *E. malliodora* and *E. camaldulensis* on *Staphylococcus aureus*. Iran J. Pharm. Res..

[108] Edward H.G.M., de Oliveira L.F.C., Quye A. (2001). Raman Spectroscopy of Coloured Resins Used in Antiquity: Dragon’s Blood and Related Substances. Spectrochim. Acta A Mol. Biomol. Spectrosc..

[109] Brophy J.J., Forster P.I., Goldsack R.J., Brynn Hibbert D. (1998). The Essential Oils of the Yellow Bloodwood Eucalypts (*Corymbia*, section *Ochraria*, Myrtaceae). Biochem. Syst. Ecol..

[110] Adeniyi C.B.A., Lawal T.O., Mahady G.B. (2009). In vitro Susceptibility of *Helicobacter pylori* to Extracts of *Eucalyptus camaldulensis* and *Eucalyptus torelliana*. Pharm. Biol..

[111] Silifat J.T., Ogunwande I.A., Olawore N.O., Walker T.M., Schmidt J.M., Setzer W.N., Olaleye O.N., Aboaba S.A. (2005). In vitro Cytotoxicity Activitie of Essential Oils of *Eucalyptus torreliana* F. v. Muell (Leaves and Fruits). J. Essent. Oil-Bear. Plants.

[112] Senadeera S.P.D., Lucantoni L., Duffy S., Avery V.M., Carroll A.R. (2018). Antiplasmodial β-Triketone-Flavanone Hybrids from the Flowers of the Australian Tree *Corymbia torelliana*. J. Nat. Prod..

[113] Sutherland M., Webb L., Wells J. (1960). Terpenoid Chemistry. III. The Essential Oils of *Eucalyptus deglupta* Blume and *E. torelliana* F. Muell. Aust. J. Chem..

[114] Lamberton J.A. (1964). The Occurrence of 5-Hydroxy-7,4’-dimethoxy-6-methylflavone in *Eucalyptus* Waxes. Aust. J. Chem..

[115] Lawal T.O., Adeniyi B.A., Adegoke A.O., Franzblau S.G., Mahady G.B. (2012). In vitro Susceptibility of *Mycobacterium tuberculosis* to Extracts of *Eucalyptus camaldulensis* and *Eucalyptus torelliana* and Isolated Compounds. Pharm. Biol..

[116] Massaro C.F., Katouli M., Grkovic T., Vu H., Quinn R.J., Heard T.A., Carvalho C., Manley-Harris M., Wallace H.M., Brooks P. (2014). Anti-Staphylococcal Activity of *C*-Methyl Flavanones from Propolis of Australian Stingless Bees (*Tetragonula carbonaria*) and Fruit Resins of *Corymbia torelliana* (Myrtaceae). Fitoterapia.

[117] Nobakht M., Grkovic T., Trueman S.J., Wallace H.M., Katouli M., Quinn R.J., Brooks P.R. (2014). Chemical Constituents of Kino Extract from *Corymbia torelliana*. Molecules.

[118] Adeniyi B., Odufowoke R., Olaleye S. (2006). Antibacterial and Gastroprotective Properties of *Eucalyptus torelliana* [Myrtaceae] Crude Extracts. Int. J. Pharmacol..

[119] Panikar S., Nanthini A.U.R., Umapathy V.R., SumathiJones C., Mukherjee A., Prakash P., Farooq T.H. (2022). Morphological, chemoprofile and soil analysis comparison of Corymbia citriodora (Hook.) KD Hill and LAS Johnson along with the green synthesis of iron oxide nanoparticles. J. King Saud Univ. Sci..

[120] Aguiar R.W., Ootani M.A., Ascencio S.D., Ferreira T.P., Dos Santos M.M., dos Santos G.R. (2014). Fumigant antifungal activity of *Corymbia citriodora* and *Cymbopogon nardus* essential oils and citronellal against three fungal species. Sci. World J..

[121] Alves T.J.S., Murcia A., Wanumen A.C., Wanderley-Teixeira V., Teixeira Á.A.C., Ortiz A., Medina P. (2019). Composition and Toxicity of a Mixture of Essential Oils Against Mediterranean Fruit Fly, *Ceratitis capitata* (Wiedemann) (Diptera: Tephritidae). J. Econ. Entomol..

[122] Benchaa S., Hazzit M., Abdelkrim H. (2018). Allelopathic Effect of *Eucalyptus citriodora* Essential Oil and Its Potential Use as Bioherbicide. Chem. Biodivers..

[123] Betts T.J. (2000). Solid Phase microextraction of Volatile Constituents from Individual Fresh *Eucalyptus* Leaves of Three Species. Planta Med..

[124] Bossou A.D., Mangelinckx S., Yedomonhan H., Boko P.M., Akogbeto M.C., De Kimpe N., Avlessi F., Sohounhloue D.C.K. (2013). Chemical Composition and Insecticidal Activity of Plant Essential Oils from Benin against *Anopheles gambiae* (Giles). Parasit. Vectors.

[125] Bouyahya A., Abrini J., Et-Touys A., Bakri Y., Dakka N. (2017). Indigenous Knowledge of the Use of Medicinal Plants in the North-West of Morocco and Their Biological Activities. Eur. J. Integr. Med..

[126] Chalchat J.M., Garry R.P., Sidibé L., Harama M. (2000). Aromatic Plants of Mali (V): Chemical Composition of Essential Oils of Four Eucalyptus Species Implanted in Mali: Eucalyptus camaldulensis, E. citriodora, E. torelliana and E. tereticornis. J. Essent. Oil Res..

[127] Chen J., Lichwa J., Ray C. (2007). Essential oils of selected hawaiian plants and associated litters. J. Essent. Oil Res..

[128] Cimanga K., Kambu K., Tona L., Apers S., De Bruyne T., Hermans N., Totté J., Pieters L., Vlietinck A.J. (2002). Correlation between chemical composition and antibacterial activity of essential oils of some aromatic medicinal plants growing in the Democratic Republic of Congo. J. Ethnopharmacol..

[129] Costa A.V., Pinheiro P.F., de Queiroz V.T., Rondelli V.M., Marins A.K., Valbon W.R., Pratissoli D. (2015). Chemical composition of essential oil from Eucalyptus citriodora leaves and insecticidal activity against Myzus persicae and Frankliniella schultzei. J. Essent. Oil-Bear. Plants.

[130] De Medici D., Pieretti S., Salvatore G., Nicoletti M., Rasoanaivo P. (1992). Chemical analysis of essential oils of malagasy medicinal plants by gas chromatography and NMR spectroscopy. Flavour Fragr. J..

[131] Ghaffar A., Yameen M., Kiran S., Kamal S., Jalal F., Munir B., Saleem S., Rafiq N., Ahmad A., Saba I. (2015). Chemical composition and in-vitro evaluation of the antimicrobial and antioxidant activities of essential oils extracted from seven eucalyptus species. Molecules.

[132] Homa M., Fekete I.P., Böszörményi A., Singh Y.R.B., Selvam K.P., Shobana C.S., Manikandan P., Kredics L., Vágvölgyi C., Galgóczy L. (2015). Antifungal Effect of Essential Oils against Fusarium Keratitis Isolates. Planta Med..

[133] Lee Y.S., Kim J., Shin S.C., Lee S.G., Park I.K. (2008). Antifungal activity of Myrtaceae essential oils and their components against three phytopathogenic fungi. Flavour Fragr. J..

[134] Manguro L.O.A., Opiyo S.A., Asefa A., Dagne E., Muchori P.W. (2010). Chemical constituents of essential oils from three eucalyptus species acclimatized in Ethiopia and Kenya. J. Essent. Oil-Bear. Plants.

[135] Mann T.S., Babu G.D.K., Guleria S., Singh B. (2013). Variation in the volatile oil composition of Eucalyptus citriodora produced by hydrodistillation and supercritical fluid extraction techniques. Nat. Prod. Res..

[136] Manzoor F., Naz N., Malik S.A., Arshad S., Siddiqui B. (2013). Chemical composition of essential oils derived from eucalyptus and lemongrass and their antitermitic activities angainst Microtermes mycophagus (Desneux). Asian J. Chem..

[137] Mohamed A.A., Behiry S.I., Younes H.A., Ashmawy N.A., Salem M.Z.M., Márquez-Molina O., Barbabosa-Pilego A. (2019). Antibacterial activity of three essential oils and some monoterpenes against Ralstonia solanacearum phylotype II isolated from potato. Microb. Pathog..

[138] Mulyaningsih S., Sporer F., Reichling J., Wink M. (2011). Antibacterial activity of essential oils from Eucalyptus and of selected components against multidrug-resistant bacterial pathogens. Pharm. Biol..

[139] Panikar S., Shoba G., Arun M., Sahayarayan J.J., Usha Raja Nanthini A., Chinnathambi A., Alharbi S.A., Nasif O., Kim H.J. (2021). Essential oils as an effective alternative for the treatment of COVID-19: Molecular interaction analysis of protease (Mpro) with pharmacokinetics and toxicological properties. J. Infect. Public Health.

[140] Rajeswara Rao B.R., Kaul P.N., Syamasundar K.V., Ramesh S. (2003). Comparative Composition of Decanted and Recovered Essential Oils of *Eucalyptus citriodora* Hook. Flavour Fragr. J..

[141] Ramezani H., Singh H.P., Batish D.R., Kohli R.K. (2002). Antifungal activity of the volatile oil of Eucalyptus citriodora. Fitoterapia.

[142] Sarma N., Gogoi R., Loying R., Begum T., Munda S., Pandey S.K., Lal M. (2021). Phytochemical composition and biological activities of essential oils extracted from leaves and flower parts of Corymbia citriodora (Hook). J. Environ. Biol..

[143] Setia N., Batish D.R., Singh H.P., Kohli R.K. (2007). Phytotoxicity of volatile oil from Eucalyptus citriodora against some weedy species. J. Environ. Biol..

[144] Sultana S., Ali M., Ansari S.H., Bagri P. (2008). The Effect of Physical Factors on Chemical Composition of the Essential Oil of Eucalyptus citriodora Hook. Leaves. J. Essent. Oil-Bear. Plants.

[145] Traoré N., Sidibé L., Figuérédo G., Chalchat J.C. (2010). Chemical composition of five essential oils of eucalyptus species from mali: E. houseana f.v. Fitzg. ex Maiden, e. citriodora Hook., e. Raveretiana f. Muell., e. Robusta Smith and e. Urophylla s.t. Blake. J. Essent. Oil Res..

[146] Vernin G.A., Parkanyi C., Cozzolino F., Fellous R. (2004). GC/MS analysis of the volatile constituents of corymbia citriodora hook. from réunion Island). J. Essent. Oil Res..

[147] Zini C.A., Augusto F., Christensen E., Caramão E.B., Pawliszyn J. (2002). SPME applied to the study of volatile organic compounds emitted by three species of Eucalyptus in situ. J. Agric. Food Chem..

[148] Zini C.A., Zanin K.D., Christensen E., Caramão E.B., Pawliszyn J. (2003). Solid-phase microextraction of volatile compounds from the chopped leaves of three species of Eucalyptus. J. Agric. Food Chem..

[149] Asante K.S., Brophy J.J., Doran J.C., Goldsack R.J., Hibbert D.B., Larmour J.S. (2001). A Comparative Study of the Seedling Leaf Oils of the Spotted gums: Species of the *Corymbia* (Myrtaceae), Section Politaria. Aust. J. Bot..

[150] Filomeno C.A., Barbosa L.C.A., Teixeira R.R., Pinheiro A.L., de Sá Farias E., de Paula Silva E.M., Picanço M.C. (2017). *Corymbia* spp. and *Eucalyptus* spp. Essential Oils Have Insecticidal Activity against *Plutella xylostella*. Ind. Crops Prod..

[151] Reyes E.I.M., Farias E.S., Silva E.M.P., Filomeno C.A., Plata M.A.B., Picanço M.C., Barbosa L.C.A. (2019). *Eucalyptus resinifera* Essential Oils have Fumigant and Repellent Action Against *Hypothenemus hampei*. J. Crop Prot..

[152] Santos P.L., Matos J.P.S., Picot L., Almeida J.R., Quintans J.S., Quintans-Júnior L.J. (2019). Citronellol, a monoterpene alcohol with promising pharmacological activities-A systematic review. Food Chem. Toxicol..

[153] Sharma R., Rao R., Kumar S., Mahant S., Khatkar S. (2019). Therapeutic potential of citronella essential oil: A review. Curr. Drug Discov. Technol..

[154] Salehi B., Upadhyay S., Erdogan Orhan I., Kumar Jugran A., LD Jayaweera S., Dias D.A., Sharopov F., Taheri Y., Martins N., Baghalpour N. (2019). Therapeutic potential of α-and β-pinene: A miracle gift of nature. Biomolecules.

[155] Bignell C.M., Dunlop P.J., Brophy J.J., Jackson J.F. (1996). Volatile Leaf Oils of Some South-Western and Southern Australian Species of the Genus *Eucalyptus* (series I). Part XIII. (Series I). (a) Series Subulatae, (b) Series Curviptera, (c) Series Contortae, (d) Series Incognitae, (e) Series Terminaliptera, (f) Series Inclusae, (g) Series Microcorythae and (h) Series Cornutae. Flavour Fragr. J..

[156] Ramezani H., Singh H., Batish D., Kohli R., Dargan J. (2002). Fungicidal Effect of Volatile Oils from *Eucalyptus citriodora* and its Major Constituent Citronellal. N. Z. Plant Prot..

[157] Salas-Oropeza J., Jimenez-Estrada M., Perez-Torres A., Castell-Rodriguez A.E., Becerril-Millan R., Rodriguez-Monroy M.A., Jarquin-Yañez K., Canales-Martinez M.M. (2021). Wound Healing Activity of α-Pinene and α-Phellandrene. Molecules.

[158] Tasdemir D., Kaiser M., Brun R., Yardley V., Schmidt T.J., Tosun F., Ruedi P. (2006). Antitrypanosomal and antileishmanial activities of flavonoids and their analogues: In vitro, in vivo, structure-activity relationship, and quantitative structure-activity relationship studies. Antimicrob. Agents Chemother..

[159] da Silva E.R., Brogi S., Lucon-Júnior J.F., Campiani G., Gemma S., Maquiaveli C.d.C. (2019). Dietary polyphenols rutin, taxifolin and quercetin related compounds target *Leishmania amazonensis* arginase. Food Funct..

[160] Carroll A.R., Avery V.M., Duffy S., Forster P.I., Guymer G.P., Watsonianone A.-C. (2013). Anti-Plasmodial β-Triketones from the Australian tree, *Corymbia watsoniana*. Org. Biomol. Chem..

[161] Solomon B., Gebre-Mariam T., Asres K. (2012). Mosquito repellent actions of the essential oils of cymbopogon citratus, cymbopogon nardus and eucalyptus citriodora: Evaluation and formulation studies. J. Essent. Oil-Bear. Plants.

[162] Dube F.F., Tadesse K., Birgersson G., Seyoum E., Tekie H., Ignell R., Hill S.R. (2011). Fresh, dried or smoked? Repellent properties of volatiles emitted from ethnomedicinal plant leaves against malaria and yellow fever vectors in Ethiopia. Malar. J..

[163] Seyoum A., Kabiru E.W., Lwande W., Killeen G.F., Hassanali A., Knols B.G.J. (2002). Repellency of live potted plants against Anopheles gambiae from human baits in semi-field experimental huts. Am. J. Trop. Med. Hyg..

[164] Dugassa S., Medhin G., Balkew M., Seyoum A., Gebre-Michael T. (2009). Field investigation on the repellent activity of some aromatic plants by traditional means against *Anopheles arabiensis* and *An. pharoensis* (Diptera: Culicidae) around Koka, central Ethiopia. Acta Trop..

[165] Seyoum A., Pålsson K., Kunga S., Kabiru E.W., Lwande W., Killeen G.F., Hassanali A., Knols B.G. (2002). Traditional use of mosquito-repellent plants in western Kenya and their evaluation in semi-field experimental huts against Anopheles gambiae: Ethnobotanical studies and application by thermal expulsion and direct burning. Trans. R. Soc. Trop. Med. Hyg..

[166] Braverman Y., Chizov-Ginzburg A., Mullens B.A. (1999). Mosquito repellent attracts *Culicoides imicola* (Diptera: Ceratopogonidae). J. Med. Entomol..

[167] Collins D.A., Brady J.N., Curtis C.F. (1993). Assessment of the efficacy of quwenling as a mosquito repellent. Phytother. Res..

[168] Curtis C., Lines J., Baolin L., Renz A. (1990). Natural and Synthetic Repellents. Appropriate Technology in Vector Control.

